# Engineering a biomimetic multiphasic suture anchor system for enhanced rotator cuff enthesis regeneration

**DOI:** 10.1126/sciadv.aea3128

**Published:** 2026-08-21

**Authors:** Zizhao Li, Se-Hwan Lee, Lin Xu, Marina Santos, Ruqiang Lu, Ellen Y. Zhang, Dong Hwa Kim, Bat-Ider Tumenbayar, Tyler E. Blanch, Jaeun Jung, Jayden Shin, Yongho Bae, Richard T. Tran, Thomas Schaer, Su Chin Heo

**Affiliations:** ^1^ McKay Orthopaedic Research Laboratory, Department of Orthopaedic Surgery, Perelman School of Medicine, University of Pennsylvania, Philadelphia, PA 19104, USA.; ^2^ Department of Bioengineering, School of Engineering and Applied Science, University of Pennsylvania, Philadelphia, PA 19104, USA.; ^3^ Department of Clinical Studies, New Bolton Center, School of Veterinary Medicine, University of Pennsylvania, Philadelphia, PA 19104, USA.; ^4^ Acuitive Technologies Inc., Allendale, NJ 07401, USA.; ^5^ Department of Pathology and Anatomical Sciences and Department of Biomedical Engineering, University at Buffalo, Buffalo, NY 14203, USA.; ^6^ Translational Musculoskeletal Research Center, Corporal Michael J Crescenz VA Medical Center, Philadelphia, PA 19104, USA.

## Abstract

The rotator cuff plays a vital role in shoulder movement and joint stability. Unfortunately, tears at the rotator cuff enthesis are common and frequently lead to retears after surgical intervention, particularly at the suture location and its anchor sites. These failures are typically due to the inability of current surgical treatments to mimic the native tissue complexity and provide the necessary metabolic, bioactive, and biophysical cues for effective enthesis regeneration. In this study, we engineered a biomimetic multiphasic scaffold system (BMS) to integrate with conventional suture anchor systems and deliver spatially organized structural and biological cues to enhance enthesis regeneration. The BMS consists of three distinct phases: Phase 1 features an aligned, nanofibrous decellularized tendon extracellular matrix (dECM) combined with “stiff” methacrylated hyaluronic acid (MeHA); phase 2 incorporates nonaligned, nanofibrous dECM with “soft” MeHA; and phase 3 uses a porous, bioenergetic, citrate-based composite scaffold for bone integration. In vitro, the BMS notably enhanced tenogenic, fibrochondrogenic, and chondrogenic differentiation, facilitating zone-specific rotator cuff enthesis regeneration. Further, in vivo, the BMS promoted successful integrative healing, forming distinct tendon, fibrocartilage, and bone regions at the repair site. This advanced multiphasic scaffold closely replicates native tissue properties, offering a promising strategy to improve rotator cuff repair. Its integration with conventional suture anchors provides an innovative design that enhances mechanical fixation and guides enthesis healing to reduce retear rates. Broadly, this platform offers a versatile solution for biointegrative repair strategies across complex soft-to-hard tissue interfaces.

## INTRODUCTION

**Fig. 1.**
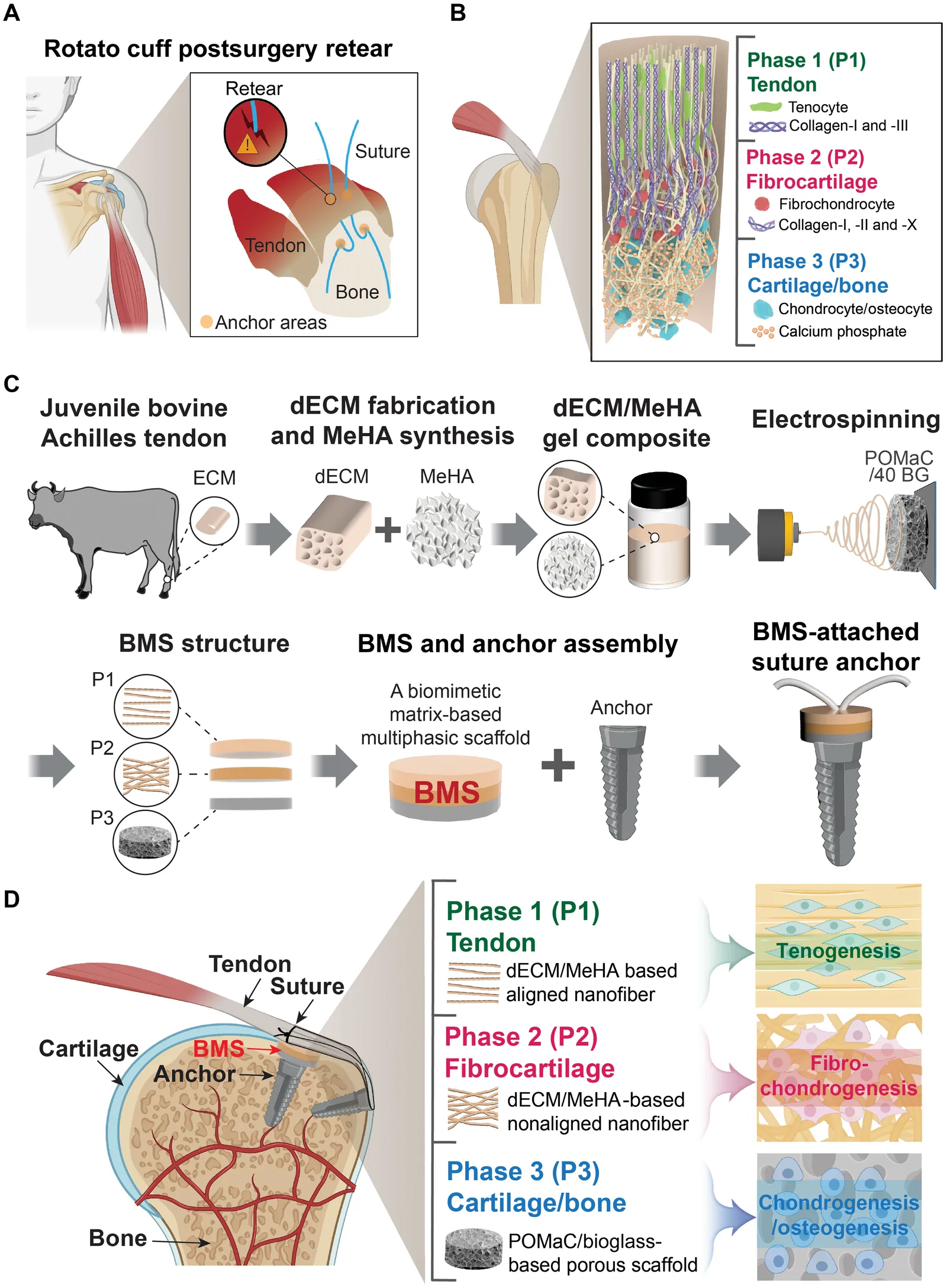
Engineering a biomimetic, multiphasic suture anchor system for enhanced rotator cuff repair. (**A**) Schematic of rotator cuff repair, emphasizing the potential for retear at suture sites postsurgery. (**B**) Structural organization of the native rotator cuff tendon-to-bone insertion (enthesis) provides insight into the suture anchor–attached BMS design. (**C**) Schematic diagram illustrating the strategy for engineering the BMS to be used with suture anchors. (**D**) Schematic illustration of the BMS scaffold integrated with suture anchors in rotator cuff repair techniques to enhance enthesis regeneration. Schematics in (A) to (D) were created in BioRender. S. C. Heo (2026) https://BioRender.com/9z6kaqr.

The rotator cuff comprises four muscles and their associated tendons which stabilize the joint and enable shoulder movement (*1*–*3*). Rotator cuff tears lead to significant pain, weakness, irritation, and diminished range of motion in affected individuals (Fig. 1A) (*1*–*3*). These injuries are particularly prevalent in the adult population, with approximately 2 million cases reported annually in the United States and 30% of adults over 60, and 62% of adults over 80, affected globally (*4*, *5*). Current arthroscopic shoulder repairs use suture anchor systems to reattach the torn tendon to the humeral head (*6*, *7*). However, these procedures often fail to restore the native enthesis structure, resulting in scar tissue formation, reduced mechanical integrity, and a high incidence of retear (Fig. 1A) (*8*, *9*). A key limitation lies in the design of conventional suture anchors, typically made of bioinert polymers which lack the biochemical and mechanical cues necessary to support enthesis regeneration (*10*, *11*). These challenges emphasize an urgent need for advanced therapeutic strategies that improve functional and durable tendon-to-bone healing.

The native rotator cuff enthesis is a complex, multitissue interface characterized by a seamless transition from tendon to fibrocartilage to bone (Fig. 1B) (*4*, *5*). The zone-dependent architecture of the enthesis is crucial for reducing stress concentrations, enabling effective load transfer, and providing mechanical stability yet remains difficult to regenerate. While recent regenerative engineering advances have led to the development of commercial scaffolds (*12*–*15*), challenges persist. Notable commercial examples include porous implants derived from bovine Achilles tendon and polylactide fiber–based scaffolds designed to support healing (*16*, *17*). However, these scaffolds often do not replicate the multitissue architecture and lack key biochemical cues, limiting their effectiveness in restoring the native enthesis. More recently, a number of interfacial tissue engineering strategies have been explored to regenerate the complex tendon-to-bone transition at the rotator cuff insertion site (*13*, *18*). These efforts include bilayered synthetic nanofiber constructs and spatially graded natural polymer systems that aim to mimic the native enthesis (*14*, *15*). While these strategies have advanced the field, current designs still often fail to simultaneously address the biological and mechanical demands for functional interface regeneration (*19*, *20*). In particular, synthetic scaffolds often provide mechanical support but lack the bioactive signals needed to promote formation of the calcified cartilage zone, whereas biologically inspired or hydrogel-based systems frequently struggle to maintain structural stability under physiological loading (*19*, *21*, *22*). Together, these limitations suggest the need for biomimetic designs that better integrate biological relevance with mechanical performance in the context of rotator cuff enthesis repair and reduce the risk of retear after surgery.

Decellularized extracellular matrix (dECM) is a versatile and promising native biomaterial for tissue-specific applications (*23*). Its natural composition provides structural integrity and essential tissue-specific biochemical cues, making it ideal for regenerative engineering strategies (*24*–*26*). Building on our previous work, which demonstrated the efficacy of dECM-based nanofibrous scaffolds for fibrous connective tissue regeneration (*27*), in this study, we have developed a significantly advanced biomimetic, dECM-based multiphasic scaffold system (BMS). This system integrates bovine Achilles tendon–derived dECM with methacrylated hyaluronic acid (MeHA) to promote tendon regeneration (*28*, *29*). The strategic incorporation of MeHA allows for precise control over the hydrogel stiffness (*28*, *30*) to support tendon and fibrocartilaginous regeneration. For fibrocartilage and bone integration, a bioenergetic and biodegradable citrate-based elastomer (*31*–*33*), which has been reported to induce osteogenic differentiation in human mesenchymal stem cells (*31*, *34*), is composited with bioactive glass (BG), known for its osteogenic potential (*35*). The citrate-based composite provides metabolic and osteogenic cues for fibrocartilage-to-bone integration within the scaffold’s multiphasic structure (Fig. 1, B and C) (*36*–*39*). The feature of this approach lies in its dual-functional capability: It provides a structurally competent, osteoconductive anchor for the bone interface while preserving a soft, bioactive niche that supports tendon regeneration. The scaffold is designed for direct integration with clinically used suture anchors, thereby addressing a longstanding challenge in rotator cuff repair, achieving stable and surgically compatible fixation, that limits the clinical effectiveness of many existing hydrogel or fiber-based systems. By spatially patterning tendon-derived dECM and MeHA alongside a citrate-based elastomeric composite reinforced with BG, this system recapitulates the key biochemical and mechanical gradients of the native enthesis (Fig. 1, B and C).

Specifically, our BMS consists of three distinct phases, each tailored to the zonal architecture of the enthesis: (i) phase 1: aligned (AL), electrospun dECM scaffolds with “stiff” MeHA for tendon regeneration, (ii) phase 2: nonaligned (NAL), electrospun dECM scaffolds with “soft” MeHA for fibrocartilaginous regeneration, and (iii) phase 3: poly[octamethylene maleate (anhydride) citrate] (POMaC)/BG porous scaffolds for fibrocartilage-to-bone integration (Fig. 1C). Comprehensive in vitro evaluations demonstrated the zonal functionality of the BMS, with each phase supporting cell morphology, differentiation, and ECM deposition consistent with its intended regenerative role. For instance, stiff AL tendon dECM nanofibrous scaffolds promoted tenogenic differentiation, while soft NAL dECM scaffolds facilitated fibrochondrogenic differentiation. In addition, POMaC/BG porous scaffolds demonstrated enhanced mineralization and chondrogenic differentiation, indicating their role in supporting the fibrocartilage-to-bone tissue transition. Further, to evaluate the translational potential of the BMS, we used a rabbit rotator cuff repair model, where native enthesis tissue was surgically removed and replaced with the BMS scaffold. In vivo results demonstrated that the BMS facilitated integrative healing, with distinct tendon, fibrocartilage, and bone zones forming at the repair site. Micro-computed tomography (micro-CT) analyses revealed enhanced bone remodeling, and histology confirmed the successful integration of the scaffold with native tissues, highlighting its potential for zone-specific rotator cuff enthesis repair.

Our multiphasic scaffold system integrates with conventional suture anchors to enhance mechanical fixation and zone-specific regeneration by providing critical biological and structural cues (Fig. 1D). This biomimetic approach mimics the native enthesis architecture, offering a more effective repair strategy and a strong foundation for future clinical studies.

## RESULTS

### Decellularized bovine ECM characterization and proteomic analysis

**Fig. 2.**
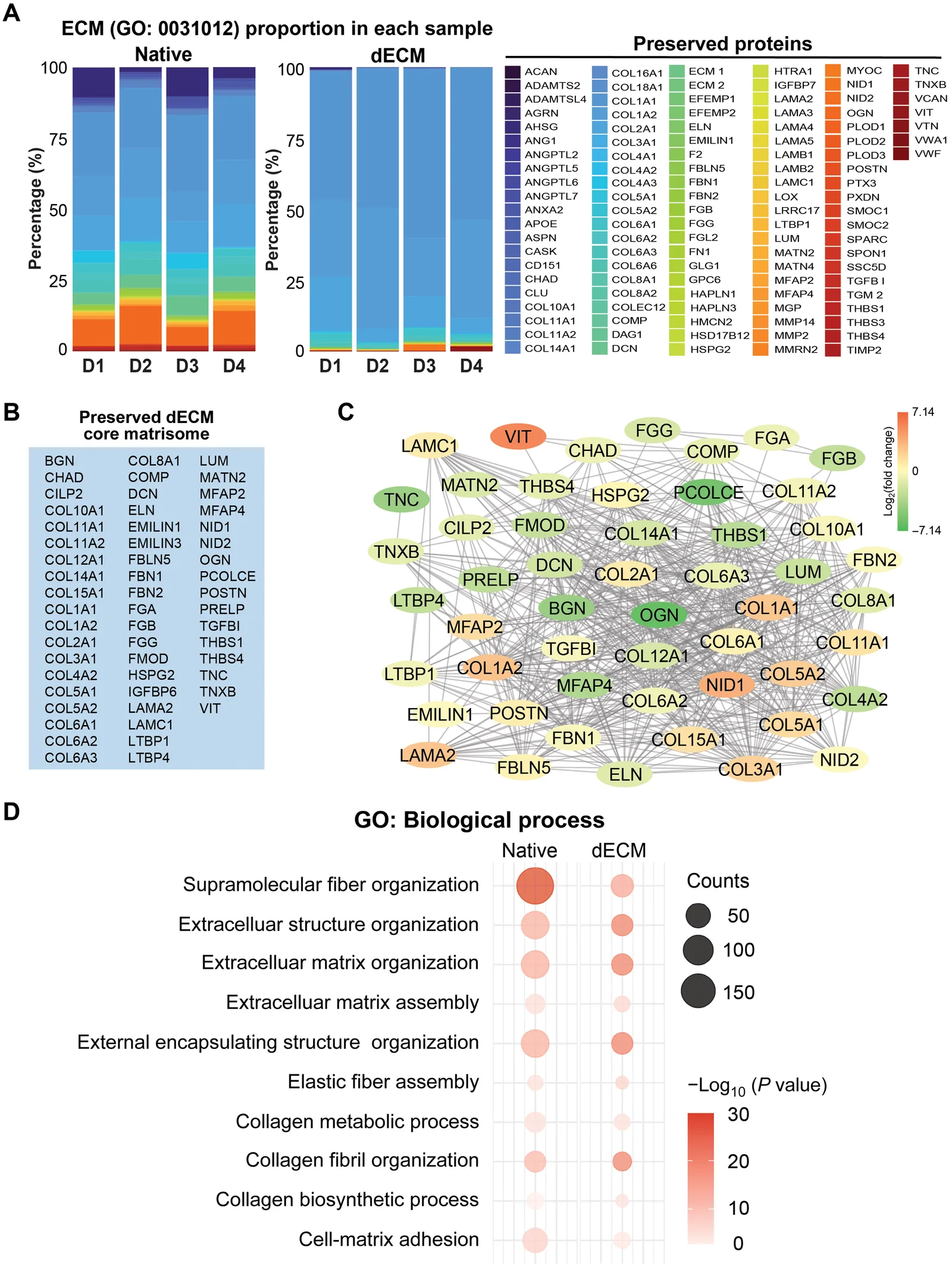
Proteomic profiling of decellularized bovine Achilles tendon ECM. (**A**) Stacked bar plots showing the composition of ECM proteins in native versus dECM samples across four donors (D, donor; donor # = 4 per group), highlighting preserved proteins postdecellularization. (**B**) List of core ECM proteins retained in dECM samples. (**C**) Protein-protein interaction network illustrating the interconnectivity of core ECM components in dECM. (**D**) GO analysis of biological process categories related to ECM structure and function.

Following the decellularization process, hematoxylin and eosin (H&E) staining confirmed successful decellularization of the bovine Achilles tendon, showing complete removal of nuclei (fig. S1A). However, key ECM proteins, specifically collagen and glycosaminoglycans (GAGs), were retained, as verified by Picrosirius Red (PSR) and Alcian Blue staining, respectively (fig. S1A). To assess ECM composition before and after decellularization, we conducted proteomic analysis on native and dECM samples. The principal components analysis demonstrated distinct clustering of native and dECM samples (fig. S1B). Proteomic analysis identified 2416 unique proteins in the native bovine Achilles tendon, with 250 proteins common to native and dECM tissues (fig. S1C). Stacked bar plots across four donors (Fig. 2A) showed overall retention of ECM proteins postdecellularization, with tendon-associated collagens [e.g., collagen type I alpha 1 chain (COL1A1), collagen type I alpha 2 chain (COL1A2), collagen type III alpha 1 chain (COL3A1), collagen type V alpha 1 chain (COL5A1), collagen type V alpha 2 chain (COL5A2), collagen type VI alpha 1 chain (COL6A1), collagen type VI alpha 2 chain (COL6A2), and collagen type VI alpha 3 chain (COL6A3)] preserved in the dECM (Fig. 2A). We further identified core matrisome components in the dECM and constructed a dense protein-protein interaction network using the Search Tool for the Retrieval of Interacting Genes/Proteins (STRING) database, comprising 52 nodes with 618 protein interactions (Fig. 2, B and C). The dECM retained key ECM proteins, including collagen subtypes (e.g., COL1A1, COL1A2, COL3A1, COL5A1, and COL6A1), glycoproteins involved in matrix binding and structural integrity [e.g., laminin subunit gamma 1 (LAMC1), latent transforming growth factor beta binding protein 1 (LTBP1), elastin (ELN), and fibronectin 1 (FN1)], proteoglycans essential for ECM remodeling and cell-matrix interactions [e.g., biglycan (BGN), decorin (DCN), and osteoglycin (OGN)], and proteins associated with ECM organization and angiogenesis [e.g., periostin (POSTN), tenascin-C (TNC), thrombospondin 4 (THBS4), and heparan sulfate proteoglycan 2 (HSPG2)], indicating preserved remodeling capacity, enhanced cell adhesion, and regenerative potential (Fig. 2, B and C). Gene ontology (GO) analysis further revealed a significant enrichment of biological processes related to extracellular structure organization, collagen biosynthesis, and cell-matrix adhesion, with comparable distributions across cellular components and molecular functions in native and decellularized tissues (Fig. 1, D and E, and 2D). These findings indicate that the decellularization process preserved essential ECM components and biological functions of the native tissue, supporting its suitability for regenerative applications.

### Development and biomechanical characterization of tunable dECM/MeHA nanofiber scaffolds

**Fig. 3.**
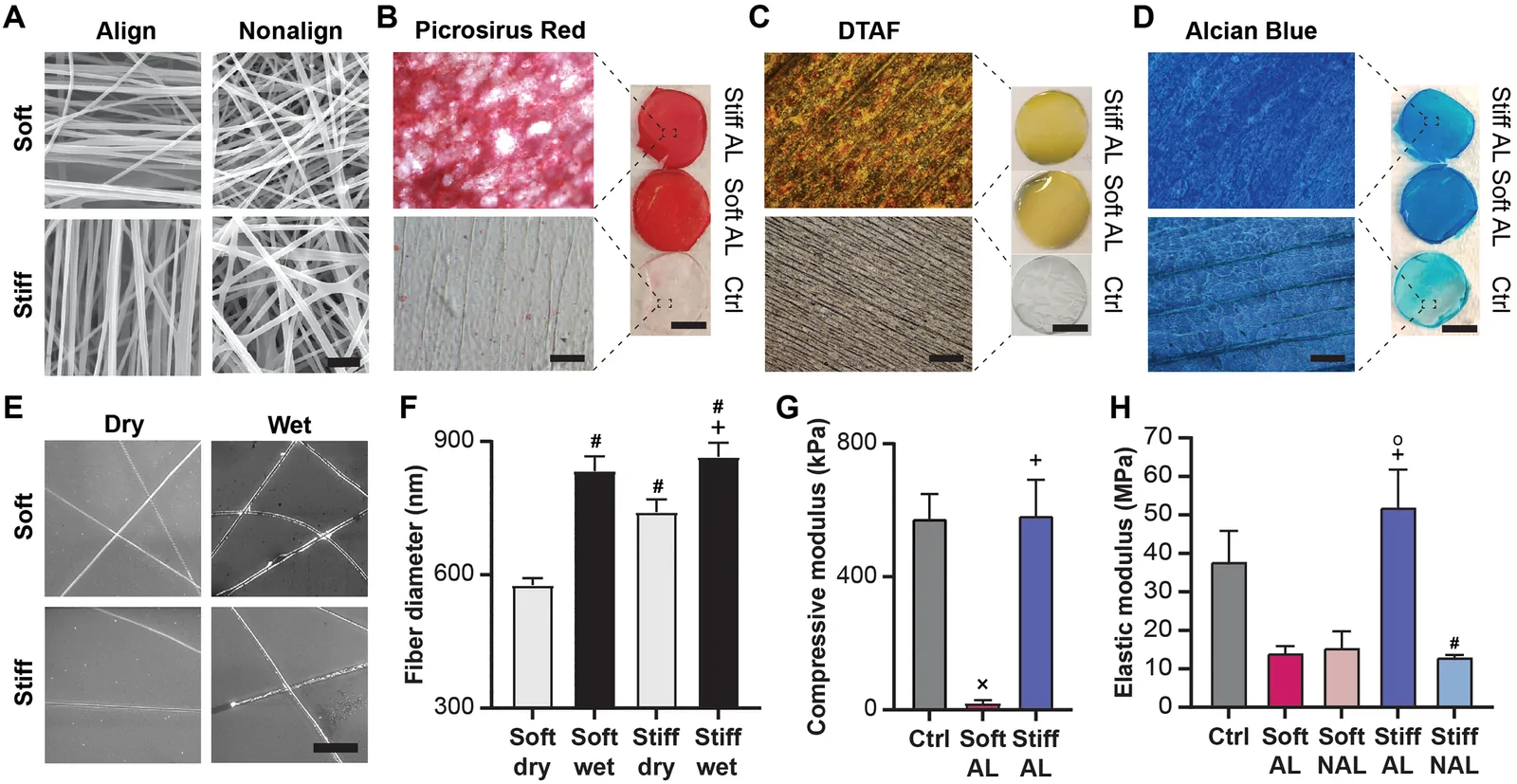
Structural and mechanical characterization of tunable tendon dECM/MeHA nanofiber scaffolds. (**A**) SEM images of soft and stiff dECM/MeHA nanofibers. Scale bar, 2 μm. (**B**) PSR staining, (**C**) DTAF staining, and (**D**) Alcian Blue staining of soft AL dECM/MeHA, stiff AL dECM/MeHA, and 100% modified AL MeHA-only control (Ctrl) nanofibers. Scale bars, 100 μm for enlarged views and 1 cm for circular scaffolds. (**E**) Representative environmental SEM (E-SEM) images of nanofibers in dry and PBS-swollen (wet) states. Scale bar, 20 μm. (**F**) Quantification of fiber diameters in dry and wet states (soft dry, soft wet, stiff dry, stiff wet; *n* = 100, #*P* < 0.0001 versus soft dry; +*P* < 0.05 versus stiff dry, means ± SEM). (**G**) Compressive modulus of Ctrl, soft AL, and stiff AL nanofibers (*n* = 27 measurements among three scaffolds per group, ×*P* < 0.0001 versus Ctrl; +*P* < 0.0001 versus soft AL, means ± SEM). (**H**) Elastic modulus of Ctrl, soft AL, soft NAL, stiff AL, and stiff NAL nanofibers (*n* = 4, ○*P* < 0.05 versus soft AL; +*P* < 0.05 versus soft NAL; #*P* < 0.05 versus stiff AL, means ± SEM).

To develop a tunable tendon dECM-based nanofibrous scaffold with adjustable fiber stiffness and alignment, MeHA was synthesized with a 35% (soft) and 100% (stiff) modification rate (*28*). These MeHA variants were blended with dECM and then electrospun and crosslinked to create AL and NAL tendon dECM-based nanofibrous scaffolds (Fig. 1C). Scanning electron microscopy (SEM) confirmed the tunable alignment of these nanofibers (Fig. 3A). Distribution and preservation of dECM within the nanofibers were qualitatively assessed using PSR, 5-(4,6-dichlorotriazinyl) aminofluorescein (DTAF), and Alcian Blue staining. Representative images of the stiff AL and control (Ctrl) groups, where the Ctrl group consisted of 100% modified MeHA without dECM, were acquired by bright-field microscopy (Fig. 3, B to D). Comparable staining patterns and matrix distribution were observed in the soft AL group.

Fiber diameters were measured in dry and phosphate-buffered saline (PBS)–swollen states using environmental SEM (E-SEM; Fig. 3E). The dry diameter of the stiff fibers was ∼28% larger than the soft fibers; however, both stiff and soft fibers showed a consistent diameter (∼850 nm) when hydrated. The morphological consistency of nanofibers ensures comparable cell-fiber interactions across groups posthydration, while stiffness remains tunable (Fig. 3F). Mechanical testing revealed the compressive modulus to be ∼29 kPa for soft fibers and ∼585 kPa for stiff fibers, with the control group showing a similar modulus (∼575 kPa) (Fig. 3G). Tensile testing further demonstrated that stiff AL scaffolds had the highest elastic modulus (∼52 MPa), nearly four times higher than other MeHA-dECM groups (Fig. 3H). These data confirm the successful fabrication of tendon dECM-based MeHA nanofiber scaffolds featuring key tendon ECM components and tunable mechanical properties, supporting their use in zone-specific tendon regeneration strategies.

### Determining scaffold stiffness and alignment to enhance zone-specific regeneration in the rotator cuff enthesis

Next, we investigated how stiffness and alignment of the dECM-based scaffold system influenced cellular phenotype, aiming to identify optimal scaffold conditions for tendon (phase 1) and fibrocartilaginous (phase 2) regeneration in rotator cuff enthesis repair. To this end, bovine mesenchymal stem cells (bMSCs) or bovine tenocytes (bTCs) were cultured on the tunable dECM-based scaffolds.

**Fig. 4.**
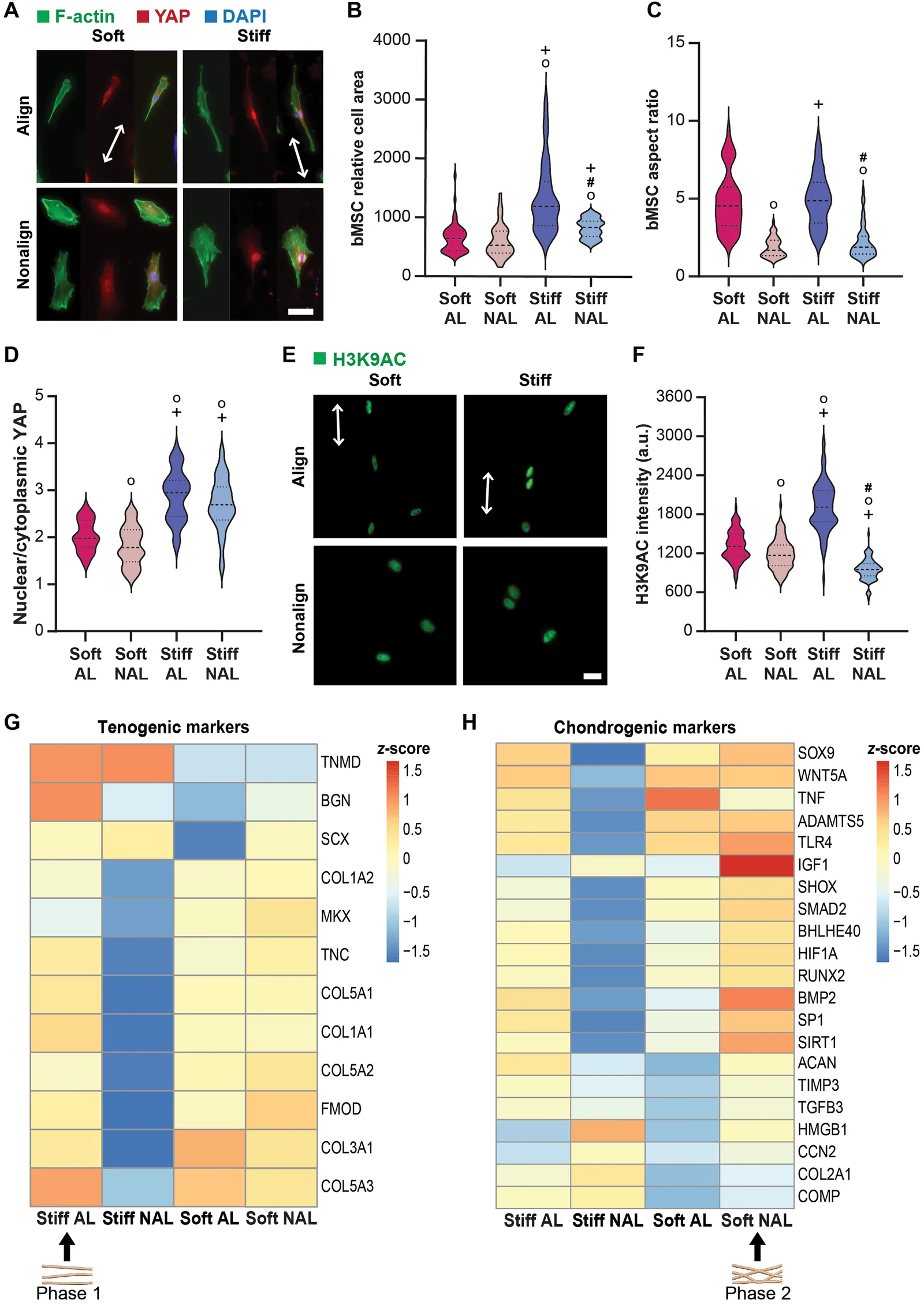
Tunable dECM scaffolds modulate cell morphology and gene expression. (**A**) Representative images of bMSCs cultured on dECM-based nanofibrous scaffolds for 3 days [arrows: fiber direction; green: F-actin, red: YAP, blue: 4′,6-diamidino-2-phenylindole (DAPI)]. Scale bar, 100 μm. Quantifications of relative (**B**) bMSC areas (*n* = 50 per group; ○*P* < 0.001 versus soft AL, +*P* < 0.01 versus soft NAL, #*P* = 0.0012 versus stiff AL, means ± SEM), (**C**) bMSC aspect ratios (*n* = 50 per group; ○*P* < 0.0001 versus soft AL, +*P* < 0.0001 versus soft NAL, #*P* < 0.0001 versus stiff AL, means ± SEM), and (**D**) YAP nuclear localization ratios (*n* = 50 per group; ○*P* < 0.05 versus soft AL, +*P* < 0.0001 versus soft NAL, means ± SD). (**E**) Representative H3K9ac fluorescence images in bMSC nuclei in cells cultured on dECM-based scaffolds after 3 days (arrows: fiber direction). Scale bar, 100 μm. (**F**) Quantification of H3K9ac fluorescence intensity in bMSC nuclei (*n* = 50 per group; ○*P* < 0.01 versus soft AL, +*P* < 0.0001 versus soft NAL, #*P* < 0.0001 versus stiff AL, means ± SD). (**G**) *Z*-score heatmaps of tenogenic gene expression via RNA-seq (*n* = 4 per group from four biological replicates). (**H**) *Z*-score heatmaps of chondrogenic gene expression via RNA-seq (*n* = 4 per group from four biological replicates). a.u., arbitrary units.

The incorporation of tendon-derived dECM components within scaffolds significantly enhanced cell attachment, even in the absence of additional cell adhesion peptides such as arginylglycylaspartic acid (RGD), compared to100% modified AL MeHA-only scaffolds (Ctrl), where cells appeared smaller, rounder, and less proliferative (fig. S2A). bMSCs and bTCs cultured on the stiff scaffolds were larger than those on the soft scaffolds, and cells on AL scaffolds exhibited higher aspect ratios than those on NAL scaffolds, indicating the capacity of these scaffolds to regulate cell attachment and shape (Fig. 4, A to C, and fig. S2, A to D). Cells exhibited high proliferation on all dECM-based scaffolds, indicating no scaffold-induced cytotoxicity (fig. S2C).

Furthermore, bMSCs seeded on stiff nanofiber scaffolds demonstrated increased nuclear localization of Yes-associated protein (YAP), a mechanosensitive transcriptional regulator, compared to those on soft scaffolds, regardless of alignment (Fig. 4, A and D). In addition, the fluorescence intensity of H3K9ac [acetylation on histone H3 lysine 9, a marker for transcriptional activation (*40*)] was significantly higher in the bMSC nuclei seeded on the stiff AL group compared to other dECM-contained scaffold groups (Fig. 4, E and F). These findings suggest that scaffold stiffness and alignment modulate epigenetic and transcriptional activity of cultured cells.

RNA sequencing (RNA-seq) analysis revealed that the stiff AL group enhanced the expression of tenogenic genes, indicating their potential suitability for promoting tendon zone (phase 1) regeneration in rotator cuff enthesis repair (Fig. 4G). In contrast, the soft NAL group promoted both chondrogenic and tenogenic gene expression, suggesting their potential for supporting regeneration of the fibrocartilaginous tendon-to-bone interface (phase 2) (Fig. 4, G and H). In addition, quantitative reverse transcription polymerase chain reaction (RT-qPCR) results showed that bTCs cultured on stiff AL highly expressed fibrous and tenogenic genes, while those on soft NAL scaffolds up-regulated chondrogenic markers (fig. S2E).

These data demonstrate that the stiffness and alignment of the dECM-based scaffold system regulate cell behavior and gene expression. The stiff AL scaffolds exhibit a more pronounced tenogenic phenotype compared to other groups, making them well-suited for tendon regeneration (phase 1), while the soft NAL scaffolds enhance both chondrogenic and tenogenic phenotypes, making them appropriate for fibro-chondrogenic differentiation (phase 2). Thus, these results indicate their potential to facilitate complex tissue integration within a BMS for rotator cuff enthesis repair.

### Development of bioactive POMaC/BG composite scaffolds for enhancing fibrocartilage-bone interface regeneration

**Fig. 5.**
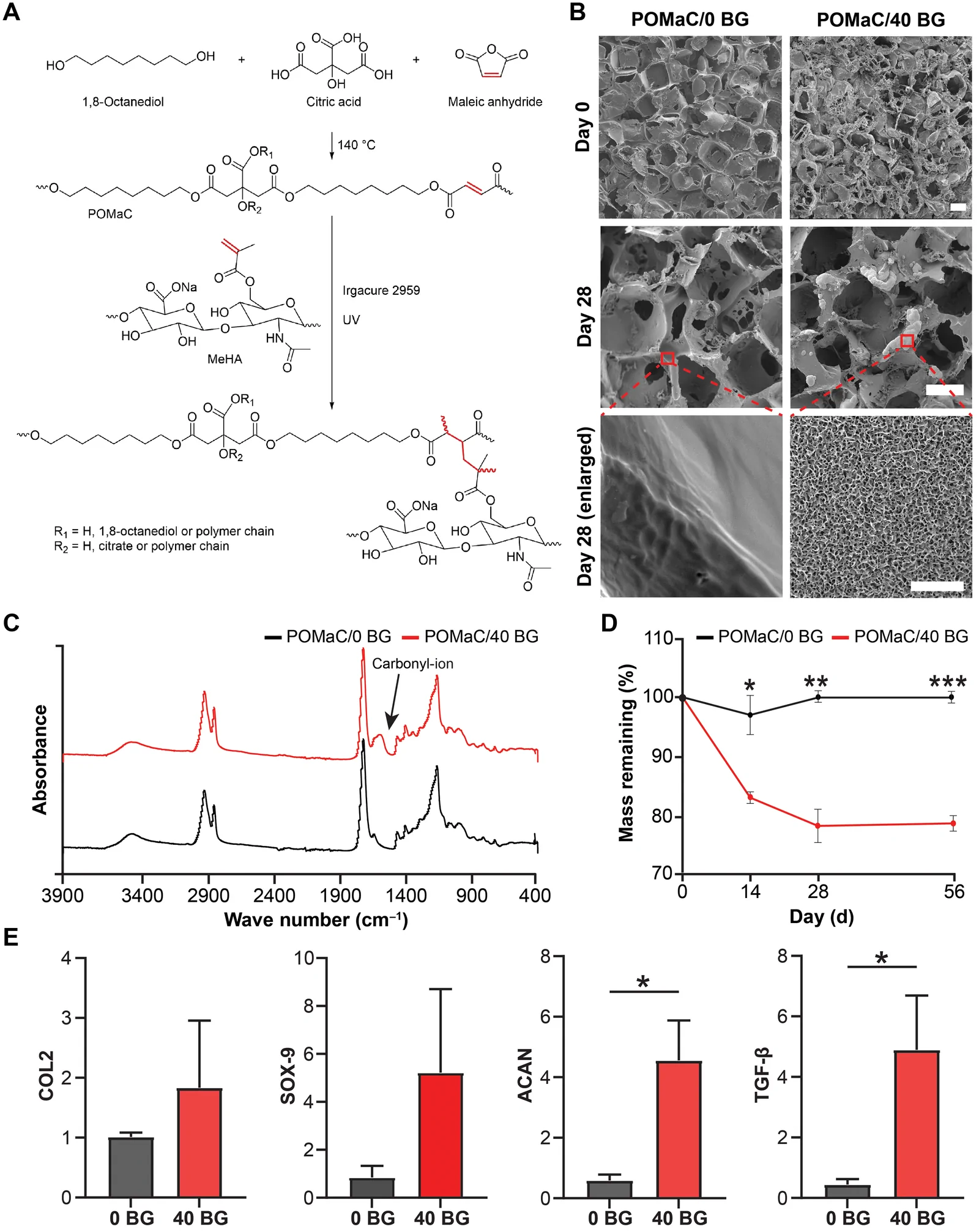
POMaC/BG scaffolds for fibrocartilaginous-bone interface regeneration. (**A**) Schematic of radical polymerization between POMaC and MeHA forming a crosslinked network. (**B**) SEM images showing surface morphology and mineralization of POMaC/0 BG and POMaC/40 BG scaffolds on day 0 and day 28. Scale bars, 200 μm for full images and 2 μm for zoomed-in images. (**C**) FTIR spectra of POMaC/0 BG and POMaC/40 BG scaffolds. (**D**) Degradation rates of both scaffolds in PBS over time (*P < 0.05, **P < 0.001, and ***P < 0.0001, *n* = 4 per group, means ± SEM). (**E**) Relative chondrogenic gene expression in bMSCs at day 14 (*P < 0.05, *n* = 5 per group, means ± SEM). *COL2*, Collagen type 2. *SOX-9*, SRY-box transcription factor 9. *ACAN*, aggrecan. *TGF-β*, transforming growth factor–β.

Since the fibrocartilaginous-to-bone region of the rotator cuff enthesis requires a biomaterial capable of supporting both mineralization and fibrocartilage-like tissue formation, we next developed a porous, composite scaffold for phase 3. This phase is composed of a citrate-based composite, POMaC, a bioenergetic molecule in bone physiology (*31*, *32*, *34*), and BG, facilitating the exchange of network modifier ions such as Na^+^ and Ca^2+^ with H^+^ (*36*, *37*, *39*, *41*). To ensure robust integration between phase 1/2 and phase 3, the dECM-based nanofibrous scaffold was directly electrospun onto the POMaC-based porous scaffold. Free radical polymerization between MeHA and POMaC at their interface can create strong adhesion between the two phases (Fig. 5A). Furthermore, hydrogen bonding between functional groups, including hydroxyl and carboxylic acid groups, present in both MeHA and POMaC further enhances this interfacial adhesion. Porous scaffolds containing 0 wt % (POMaC/0 BG) and 40 wt % BG (POMaC/40 BG) were fabricated via salt leaching, resulting in comparable pore sizes (355 to 500 μm) between groups (Fig. 5B and fig. S3A). Thermogravimetric analysis confirmed ∼40% BG incorporation in POMaC/40 BG (fig. S3B). Fourier transform infrared spectroscopy (FTIR) validated the interaction between POMaC and BG. Compared to POMaC/0 BG, POMaC/40 BG showed a distinct peak at 1599 cm^−1^ (Fig. 5C), indicative of metallic carboxylate formation (sodium/calcium carboxylates or calcium dicarboxylate bridge) in the composite (*42*). This suggests that BG acts as a filler and a crosslinking agent within the composite. Incorporating BG into POMaC accelerated the degradation rate of the scaffolds, with 21.7% mass loss after 56 days in PBS (Fig. 5D). In comparison, POMaC/0 BG exhibited less than 1% mass loss, suggesting that BG composites can modulate scaffold biodegradability. POMaC/0 BG and POMaC/40 BG scaffolds exhibited a three-phase swelling: an initial rapid absorption phase, followed by a slower swelling rate, culminating in a stable plateau (fig. S3C). Notably, the POMaC/40 BG scaffold exhibited a lower swelling than POMaC/0 BG at day 1 and day 4, contributing to scaffold structural integrity (fig. S3C).

Assessing the ability of an implantable material to induce apatite formation in simulated body fluid (SBF) is crucial for predicting its in vivo bone interactions (*43*). Apatite, a calcium and phosphate-rich mineral, facilitates biomaterial bonding to native bone (*44*). A biomaterial is deemed bioactive if apatite precipitates on its surface within a defined period during SBF incubation. After 28 days in SBF, POMaC/0 BG scaffolds showed no obvious mineralization (Fig. 5B, day 28). In contrast, POMaC/40 BG scaffolds exhibited significant apatite formation, indicating enhanced bioactivity (Fig. 5B, day 28). Cultures of bMSCs on both POMaC/0 BG and POMaC/40 BG scaffolds exhibited significant cell proliferation, indicating no cytotoxicity from the scaffold components (fig. S3D). Furthermore, bMSCs cultured on POMaC/40 BG showed significantly higher expression of chondrogenic markers [e.g., collagen type 2 (*COL2*), SRY-box transcription factor 9 (*SOX-9*), aggrecan (*ACAN*), and transforming growth factor–β (*TGF-β*)] compared to those on POMaC/0 BG, suggesting enhanced chondrogenic differentiation due to BG addition (Fig. 5E).

These data indicate that integrating BG within POMaC scaffolds enhances mineralization, stability, and degradation. In addition, the elevated chondrogenic gene expression observed in POMaC/40 BG scaffolds indicates a microenvironment favorable for fibrocartilage-like differentiation, supporting its use within the calcified zone of the tendon (phase 3) of the BMS. These findings suggest the potential of citrate-based biocomposite scaffolds to address the multifaceted requirements of enthesis regeneration by simultaneously promoting mineralization and creating an environment conducive to fibrocartilaginous tissue development.

### Development and evaluation of a BMS for zone-specific regeneration of the rotator cuff enthesis

**Fig. 6.**
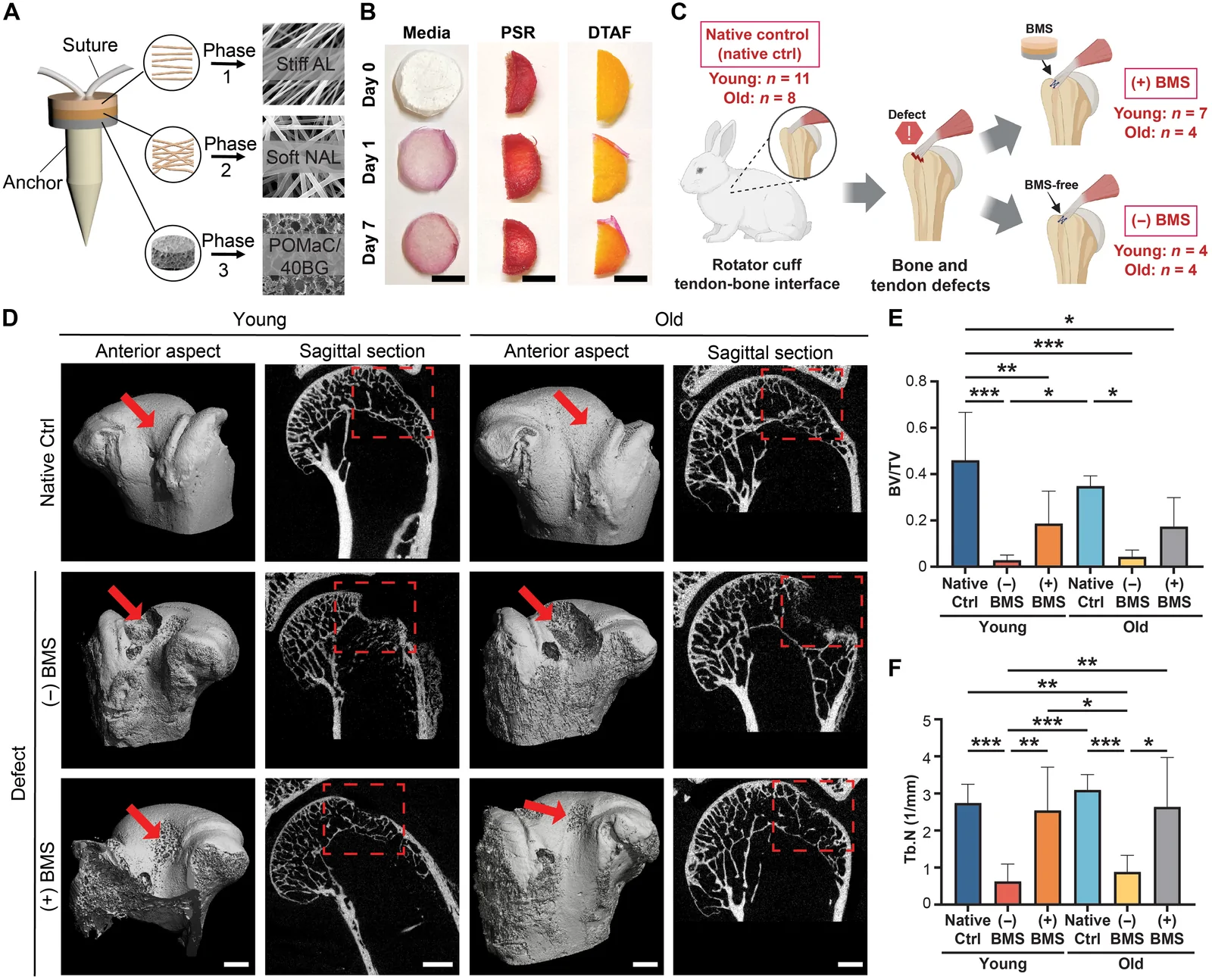
Micro-CT evaluation of the BMS in rotator cuff repair. (**A**) Schematic of BMS assembly illustrating the three-phase design for zone-specific regeneration of the rotator cuff enthesis. (**B**) Evaluation of structural stability and protein retention in the assembled BMS scaffold using PSR and DTAF staining. Scale bars, 50 mm. (**C**) Schematic of the rabbit rotator cuff repair model and surgical procedure, including scaffold placement and experimental groups: native Ctrl (native control; young: *n* = 11, old: *n* = 8), [(−) BMS] (suture-repaired control without BMS scaffold implantation; young: *n* = 4, old: *n* = 4), and [(+) BMS] (suture repair with BMS scaffold implantation; young: *n* = 7, old: *n* = 4). “Young” refers to rabbits <10 months old; “old” refers to rabbits >3 years old. (**D**) Representative micro-CT images at 4 weeks postsurgery showing bone healing across native Ctrl, [(−) BMS], and [(+) BMS] groups, with highlighted regions of interest. (**E**) Quantitative micro-CT analysis of bone remodeling, including bone volume/total volume (BV/TV) and (**F**) trabecular number (Tb.N). Significant differences among groups are indicated (*P < 0.005, **P < 0.001, and ***P < 0.0001, means ± SD). Schematic in (C) was created in BioRender. S. C. Heo (2026) https://BioRender.com/a66g22z.

We identified the optimal scaffold compositions for each regenerative zone within the BMS by building on extensive in vitro evaluations of dECM-based nanofibrous scaffolds and POMaC-based porous scaffolds. Specifically, stiff AL dECM-based nanofibrous scaffolds were selected for phase 1 (tendon regeneration), soft NAL dECM-based nanofibrous scaffolds for phase 2 (fibrocartilaginous regeneration), and POMaC/40 BG porous scaffolds for phases 3 (fibrocartilage-bone regeneration) to promote the spatially stratified regeneration of the rotator cuff enthesis. To construct the BMS, phase 1 and phase 2 dECM-based nanofibrous structures were directly electrospun onto the POMaC/40 BG porous structure (Fig. 6A). To evaluate interfacial integrity and scaffold stability, the assembled BMS was incubated in basal media for up to 7 days and assessed by gross morphological observation, PSR staining, and DTAF staining at days 0, 1, and 7. No visible delamination or phase separation was observed during the incubation period, and both PSR and DTAF staining demonstrated preservation of scaffold architecture and dECM distribution, confirming maintenance of structural integrity and protein retention within the assembled BMS construct (Fig. 6B).

Next, to assess zone-specific tissue regeneration and integrative rotator cuff healing, the BMS was evaluated in proof-of-concept rotator cuff model in young and old female rabbits (*45*, *46*). BMS scaffolds were placed between the tendon and bone [BMS-treated group: (+) BMS] (Fig. 6C). Control groups included untreated rabbit shoulders (native Ctrl) and injured shoulders with only suture fixation, representing the defect group without BMS scaffolds [(−) BMS] (Fig. 6C). The (+) BMS group included seven young and four adult right shoulders; the (−) BMS group included four young and four adult right shoulders. Left shoulders served as native controls (Fig. 6C).

All animals completed the study without adverse events. At 4 weeks postoperative, shoulders were harvested and processed for ex vivo analyses: Micro-CT imaging revealed enhanced bone remodeling and regeneration in the (+) BMS groups compared to the (−) BMS groups (Fig. 6D and fig. S4A). Quantitative analysis showed significantly higher bone volume/total volume (BV/TV) and trabecular number (Tb.N) in the (+) BMS group, approaching values observed in native controls (Fig. 6, E and F). In addition, trabecular separation (Tb.Sp) was significantly reduced in the (+) BMS group compared to the (−) BMS group (fig. S4B). Notably, both young and adult rabbits treated with BMS scaffolds demonstrated similar improvements in bone regeneration, indicating that scaffold efficacy was consistent across age groups.

**Fig. 7.**
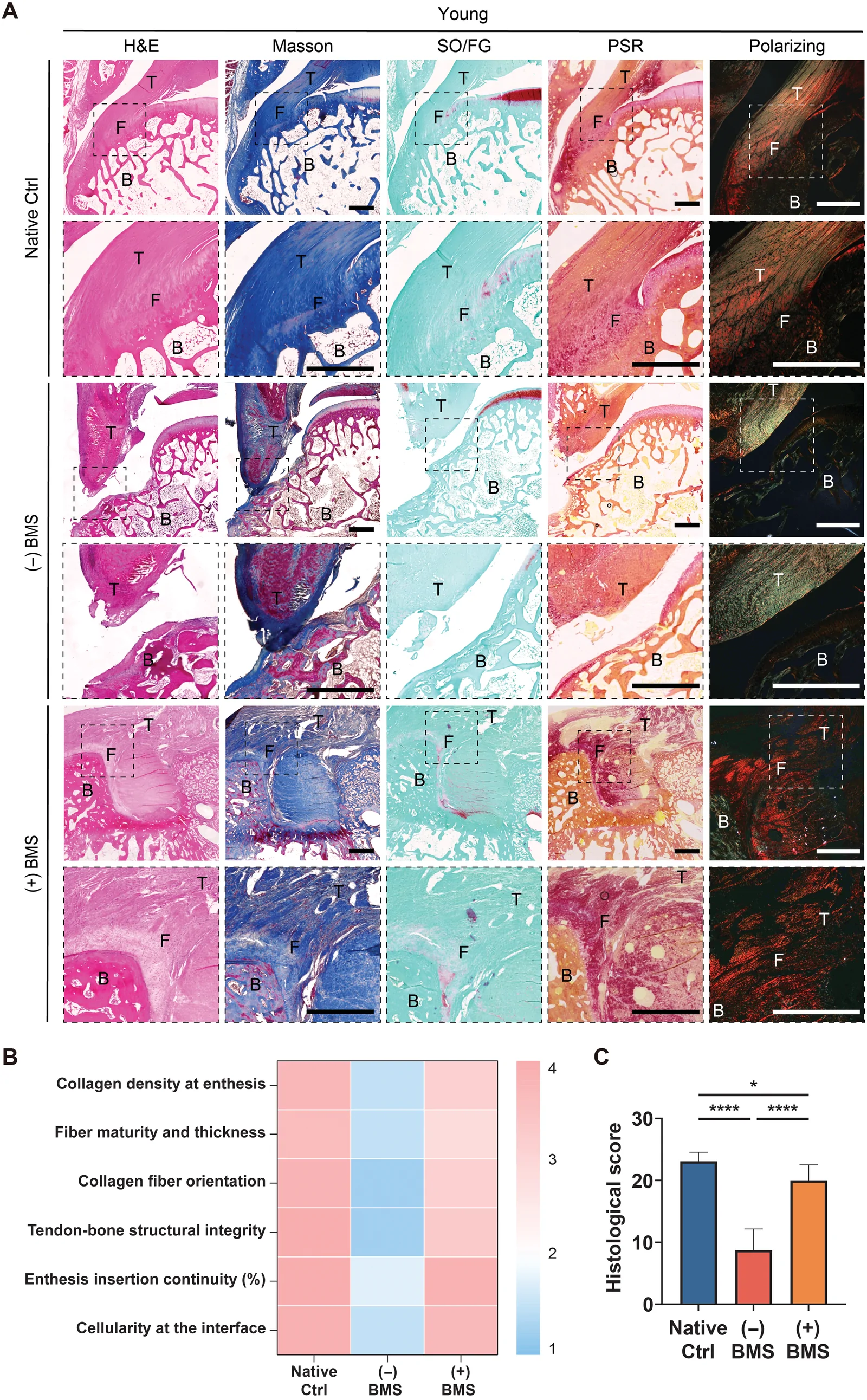
In vivo histological assessment of rotator cuff repair using the BMS in young rabbits. (**A**) Representative histological images of the tendon-to-bone interface at 4 weeks postoperation, stained with H&E, Masson’s trichrome (Masson), Safranin O/Fast green (SO/FG), and PSR, with corresponding polarized light micrographs. H&E, Masson, SO/FG, and PSR were captured by a Zeiss Axiocam 506. The polarized light micrographs were captured by a Leica DMLP Polarizing Light Microscope. The (+) BMS group shows well-organized tissue architecture with distinct tendon, fibrocartilage, and bone layers, indicating successful integration and zonal regeneration in young rabbits. Scale bars, 1 mm. T, tendon; FC, fibrocartilage; B, bone. (**B**) Heatmap summarizing histological scoring parameters across groups. (**C**) Quantitative analysis of total histological scores for tendon-enthesis units, demonstrating significantly improved outcomes in the (+) BMS group compared to controls (*P < 0.005 and ****P < 0.00001, means ± SD).

**Fig. 8.**
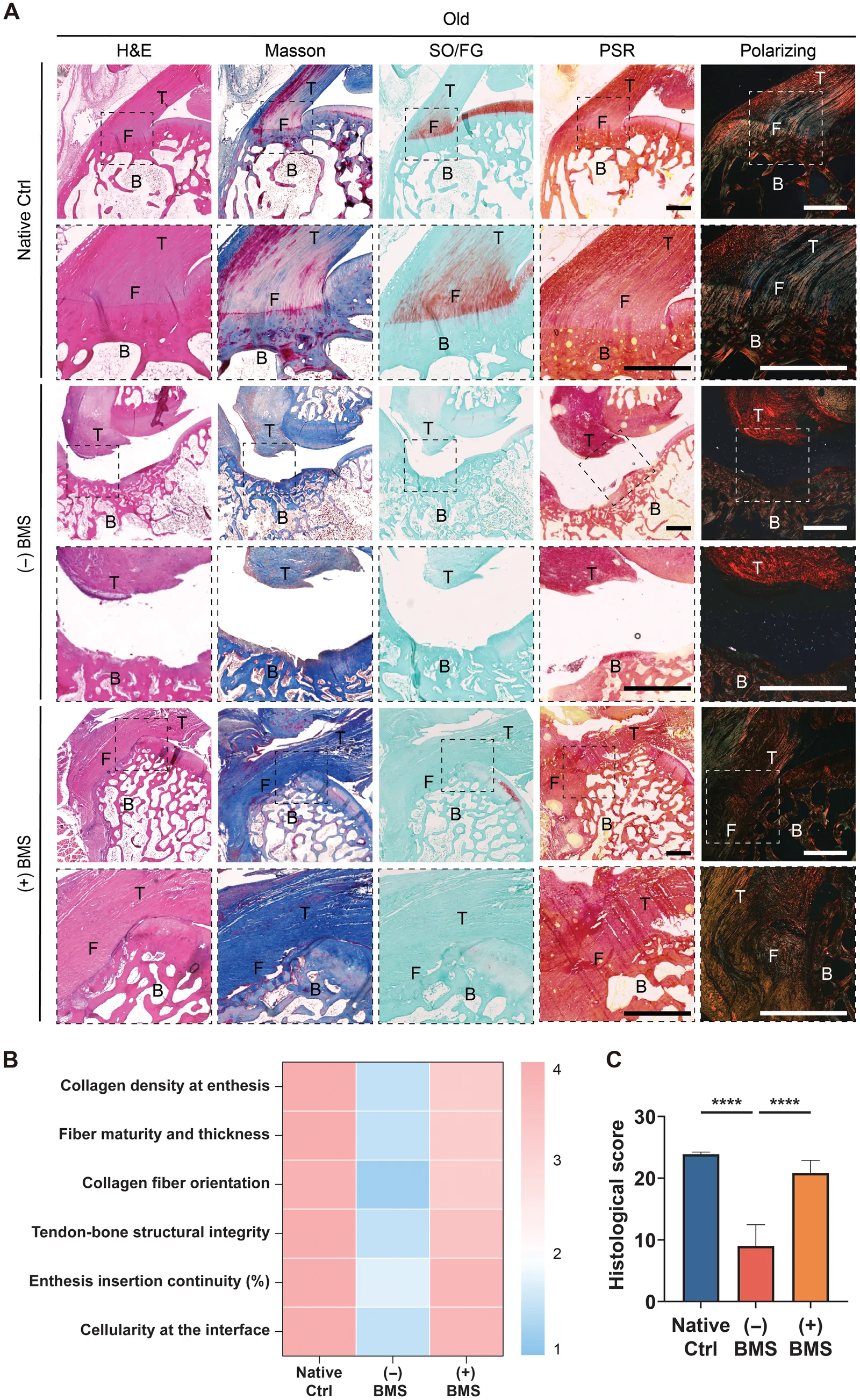
In vivo histological assessment of rotator cuff repair using the BMS in old rabbits. (**A**) Representative histological images of the tendon-to-bone interface at 4 weeks postoperation, stained with H&E, Masson’s trichrome (Masson), Safranin O/Fast green (SO/FG), and PSR, with corresponding polarizing light micrographs. H&E, Masson, SO/FG, and PSR were captured by a Zeiss Axiocam 506. The polarized light micrographs were captured by a Leica DMLP Polarizing Light Microscope. The (+) BMS group shows well-organized tissue architecture with distinct tendon, fibrocartilage, and bone layers, indicating successful integration and zonal regeneration in old rabbits. Scale bars, 1 mm. (**B**) Heatmap summarizing histological scoring parameters across groups. (**C**) Quantitative analysis of total histological scores for tendon-enthesis units, demonstrating significantly improved outcomes in the (+) BMS group compared to controls (****P < 0.00001, means ± SD).

Next, to evaluate the integration of the BMS with native rotator cuff tissues, histology using H&E, Masson’s trichrome, Safranin O/Fast Green, and PSR staining in young and old (−) BMS groups was characterized by disorganized tissue architecture, absence of fibrocartilage, and disrupted tendon-bone continuity. The insertion site exhibited immature tendon tissue formation and degenerated bone morphology, indicating poor repair (Figs. 7 and 8). In contrast, the (+) BMS groups showed substantially improved healing responses, with well-organized matrix formation and structural regeneration at the tendon-bone interface (Figs. 7 and 8 and fig. S5). In particular, the old (+) BMS group also exhibited a well-defined gradient structure, with distinguishable tendon, fibrocartilage, and bone regions at the enthesis, which were absent in the corresponding (−) BMS groups (Fig. 8). To quantitatively assess tissue regeneration, we applied a modified histological scoring system focused on collagen content, fiber organization, insertion continuity, and cellularity (table S2). At 4 weeks, both young and old (+) BMS groups achieved significantly higher histological scores compared to their (−) BMS counterparts, approaching levels observed in native controls (Figs. 7, B and C, and 8, B and C). These findings reflect enhanced collagen deposition, improved fiber alignment, greater enthesis insertion integrity, and increased cellular presence, indicating that BMS implantation promotes robust and zone-specific regeneration of the rotator cuff enthesis. Moreover, this approach offers a promising solution to overcome the limitations of conventional suture anchors, which lack the biological cues and structural guidance necessary for enthesis regeneration.

## DISCUSSION

Rotator cuff injuries are prevalent, and the gold standard repair method is suturing using hard polymer suture anchors (*1*–*3*). Unfortunately, retears occur frequently, with rates as high as 90% (*8*, *9*). This is often due to stress concentrations in these repaired regions (*8*, *9*). From a biomaterial perspective, commercial suture anchor systems focus solely on fixation to bone. However, the rotator cuff enthesis is a highly complex tissue that consists of roughly three phases: highly AL tendon, a fibrocartilaginous intermediate region, and cartilage-bone (*4*, *5*). Thus, a new system that recapitulates the complex enthesis tissue structure would afford an enhanced healing process. To this end, this study addresses the critical limitations of current repair strategies by introducing a tissue-engineered multiphasic scaffold system that recapitulates the structural, mechanical, and biochemical properties of the native enthesis. This scaffold system provides biomimetic properties to current suture anchor systems, enhancing zonal development and regeneration and potentially reducing retear rates.

To address these unmet clinical needs and overcome the existing method limitations, we developed a tunable, biomimetic matrix-based multiphasic scaffold (BMS) through rigorous biological evaluations. In this study, our approach produced a stiff AL tendon dECM-based and soft NAL dECM-based nanofibrous scaffold system for regenerating the tendon (phase 1) and fibrocartilage (phase 2) phases, respectively. In addition, a porous scaffold composed of POMaC (*31*–*33*) and BG (*35*) facilitated regeneration of the fibrocartilage-bone phase (phase 3). The BMS can be customized in size and delivered with traditional suture anchors, potentially providing essential bioactive and biophysical cues for effective enthesis tissue regeneration.

We selected decellularized bovine Achilles tendon as the base material, confirming that decellularization retained essential tendon matrix proteins, including collagen types I, II, III, V, and XI, biglycan, decorin, and fibromodulin. These proteins are pivotal for ECM structural support and organization, ensuring a conducive microenvironment for cellular adhesion, proliferation, and differentiation (*47*–*50*) in tendons. Although proteomic analyses confirmed preservation of key extracellular matrix proteins following decellularization, future quantitative assessments of protein abundance and compositional changes may provide additional insight into the influence of matrix composition on scaffold bioactivity and regenerative performance. We combined this material with stiff or soft MeHA (*28*, *29*) to create a tunable nanofibrous scaffold system using electrospinning and ultraviolet (UV) photocrosslinking, maintaining the dECM protein content while allowing for uncoupled stiffness and alignment modulation. The precise modulation of scaffold stiffness through MeHA incorporation is critical for mimicking the zonal architecture of the enthesis and regulating zone-dependent cell behavior and differentiation.

In vitro studies demonstrated the ability of the BMS to regulate cell behavior and gene expression in a zone-dependent manner. The stiff AL dECM scaffolds promoted tenogenic differentiation, which is evident from the increased expression of tendon-specific markers and activation of transcriptional regulators (e.g., enhanced YAP nuclear localization and H3K9ac intensity). Conversely, the soft NAL scaffolds facilitated chondrogenic and tenogenic gene expression, supporting their role in regenerating the fibrocartilage interface. These findings are consistent with our prior works and findings by Xia *et al.* (*27*) and Li *et al.* (*51*), which showed the critical roles of mechanical cues and ECM alignment in directing cell differentiation and tissue formation within the rotator cuff enthesis. Consistent with these studies, our findings reinforce the idea that mechanical cues and structural organization need to act together to support functional interface regeneration.

For enhanced fibrocartilage and bone integration (phase 3), we developed a base structure of porous citrate-based material with enhanced osteogenic potential (*31*, *33*). Recent studies have shown reduced citrate levels in injured rotator cuff tissues, impairing essential cellular metabolism for tendon regeneration (*11*). In addition, building on studies by Tran *et al.* (*31*, *32*), we used a citrate-based polymer, POMaC, to address local metabolic demands at the healing interface by supplying citrate to support cellular energetics and matrix formation, thereby enhancing tendon-bone regeneration and functional repair outcomes in rotator cuff repair. Incorporation of BG within the POMaC phase further promoted osteogenic activity and osteoconduction, consistent with previous studies (*35*, *51*, *52*). The scaffold’s porous structure, particularly at a 40% BG concentration, enabled significant mineralization, critical for osteoconduction and effective bone regeneration. While the current in vitro studies demonstrated phase-specific cellular responses through lineage-associated gene expression analyses, future investigations incorporating protein-level characterization and extracellular matrix deposition analyses will be important for further validating tendon-, fibrocartilage-, and mineralized fibrocartilage-like tissue formation within each scaffold phase.

Furthermore, the in vivo rabbit rotator cuff repair model demonstrated the translational promise of the BMS design. The in vivo tests confirmed that the scaffold facilitated integrative healing, with distinct zones of tendon, fibrocartilage, and bone forming at the repair site. Micro-CT imaging revealed enhanced bone remodeling at the fibrocartilage-to-bone interface as indicated by increased BV/TV and Tb.N alongside reduced Tb.Sp. Thus, these findings suggest that the (+) BMS group exhibited superior bone remodeling compared to the (−) BMS group featuring suture fixation alone. However, despite these improvements, the bone remodeling metrics in the (+) BMS group remained lower than those observed in the native Ctrl, indicating that full restoration of native bone structure has yet to be achieved; given the relatively short survival period, this is not unexpected. Notably, the BMS performed similarly in both young and old donors, suggesting that its regenerative capabilities are not significantly affected by donor age, making it applicable across a broad range of patients regardless of age-related tissue properties.

Histological analysis further revealed that implantation of the BMS significantly improved structural regeneration at the tendon-bone interface compared to suture-only repairs in both young and old rabbits. The clear demarcation of tendon, fibrocartilage, and bone zones in the BMS-treated tissues suggests that the scaffold’s spatially defined architecture and material composition supported zone-specific tissue remodeling. Particularly in older animals, where regenerative capacity is typically diminished, the BMS scaffold still promoted enthesis reconstruction with structural features resembling healthy native tissue. These findings indicate a critical design advantage of our approach. Prior studies have largely focused on synthetic or commercially available bovine scaffolds, which often fail to replicate the multitissue architecture of the native enthesis (*17*, *18*). In contrast, our data offer a new perspective by emphasizing the importance of preserving not only native biological and biochemical cues but also spatially defined mechanical gradients to support effective tissue integration. Together, these results suggest that future tissue engineering strategies should move beyond simple mechanical fixation and instead prioritize biomimetic designs that actively recapitulate the complex structural and developmental signals of the tendon-to-bone interface.

To translate this potential into clinical efficacy, further rigorous evaluations are still required for future studies. Although the in vivo results of this proof-of-concept study support the feasibility of phase-distinct enthesis regeneration using the BMS, the rabbit model used represents an acute repair environment with assessments limited to 4 weeks postoperatively. In contrast, clinical rotator cuff disease often involves chronic injury and repeated degeneration over extended periods. In addition, the in vivo study design did not include an anchor-only control group, which limits our ability to fully distinguish the specific biological effects of the BMS scaffold from those associated with fixation alone. While the primary objective of this proof-of-concept study was to evaluate the regenerative potential of the integrated BMS platform, future studies incorporating anchor-only controls and biomechanical assessments will be important for defining the independent and potentially synergistic contributions of scaffold augmentation and fixation to tendon-bone healing and enthesis regeneration.

Furthermore, the current study was limited to a single early postoperative time point and relatively small animal cohorts, which may not fully capture the long-term maturation, durability, and functional performance of the regenerated tendon-bone interface. In addition, donor-related variables, including age and biological sex, as well as differences in fixation strategies, may influence tissue composition, cellular behavior, and repair outcomes. Future studies incorporating extended postoperative time points, chronic injury models, larger animal cohorts, and comprehensive biomechanical, molecular, and functional evaluations will be important to further establish the efficacy and translational potential of the BMS platform. Last, systematic evaluation of the long-term mechanical durability of the BMS under physiological loading conditions, along with validation in larger animal models, will be critical for establishing the translational potential of this platform.

The translational adaptability of the BMS may extend beyond integration with suture anchor–assisted repair. Owing to its scalable and customizable design, enabled by the fabrication strategies of both the nanofibrous and porous scaffold phases, the overall construct can be adapted to provide broader coverage of the tendon-bone healing footprint (*53*, *54*). This flexibility suggests potential application of the BMS as a patch augmentation strategy for rotator cuff repair, which may further support tendon-bone integration by increasing the area exposed to regenerative cues and facilitating more uniform load transfer across the repair site (*55*, *56*). Future studies will be required to evaluate the feasibility and therapeutic efficacy of these configurations.

In conclusion, this study presents a promising multiphasic scaffold system that preserves tendon-derived bioactive proteins and elicits favorable changes in cellular behavior. The scaffold system significantly enhanced the tendon-bone interface in a proof-of-concept rabbit model, effectively mimicking the histological characteristics of native tissue. This BMS may address the limitations of current repair strategies using suture anchors alone and demonstrates significant potential for zone-specific tissue regeneration. Beyond rotator cuff applications, the principles shown in this study could be extended to other orthopedic interfaces, such as ligament and meniscus repair. The modularity of the BMS design allows for customization for various tissue interfaces, broadening its potential impact in musculoskeletal soft tissue regeneration.

## MATERIALS AND METHODS

### Decellularized bovine Achilles tendon ECM preparation

The dECM matrix was prepared according to a previously established protocol (*27*). Briefly, juvenile bovine Achilles tendon tissue (approximately 3-month-old juvenile bovine donors, Research 87) was harvested and cut into small cubes, each with a side length of 1 mm. These tissue cubes were stirred and incubated in a solution of 1% SDS/PBS (w/v) (Invitrogen; Gibco) for 3 days at room temperature, with the solution being refreshed daily. The tissue samples were then washed with 0.1% EDTA/PBS (w/v) (Thomas Scientific; Gibco) solution for 1 day at room temperature. After an overnight rinse in deionized water (DI water) at room temperature, the tissue samples were further lyophilized in a freezer dryer (Labconco) for 3 days and stored frozen at −20°C for long-term preservation.

### MeHA synthesis

MeHA was synthesized following previously published protocols with mechanical properties modulated by controlling the degree of methacrylation through adjustment of the initial methacrylic anhydride concentration (*28*, *57*). Using this approach, we generated MeHA formulations with defined degree of methacrylation corresponding to distinct stiffness regimes: soft MeHA (∼35% methacrylation) and stiff MeHA (∼100% methacrylation) (*28*, *58*). Sodium hyaluronate (Lifecore, 60 kDa) was dissolved in DI water overnight and reacted with calculated methacrylic anhydride (Sigma-Aldrich) on ice for 1.5 days. The pH of the solution was maintained between 8.5 and 9.5 by adding 5 M NaOH (Sigma-Aldrich) dropwise. The MeHA solution was dialyzed in DI water at room temperature for 14 days and then frozen at −80°C for 3 hours. Last, the MeHA was lyophilized in a freezer dryer (Labconco) for 3 days. The freeze-dried dECM was ground into powder and stored at −20°C for subsequent experiments.

### Proteomic analysis

Lyophilized native and dECM bovine tissue powders were submitted to the Proteomics Core Facility at the Children’s Hospital of Philadelphia Research Institute for liquid chromatography–tandem mass spectrometry analysis (*57*). Data processing and statistical analysis were conducted using the Spectronaut Pulsar and Perseus software platforms (Max Planck Institute). To identify ECM components, the GO term (GO:0031012 Extracellular Matrix) specific to *Bos taurus* organism was used, along with the MatrisomeDB1 ECM-protein knowledge database to delineate the core matrisome preserved in the dECM group. Protein-protein interaction network of the preserved core matrisome in dECM was constructed using STRING web tool (version 10.0, String Consortium 2020) and visualized with Cytoscape software (version 3.10.3). GO analysis was performed using gProfiler, with biological processes, cellular components, and molecular functions classified as significant with Benjamini-Hochberg corrected *P* value < 0.05. For visualization, stacked bar plots and bubble plots were generated using the ggplot2 package in R programming (*59*).

### Fabrication of dECM-based nanofibrous scaffolds

The stiff AL dECM-based nanofibrous scaffold electrospinning solution was prepared from 4% (w/v) stiff MeHA, 2% (w/v) dECM, 2% (w/v) poly(ethylene oxide) (PEO; Acros Organics, 900 kDa), and 0.5% (w/v) Irgacure 2959 (Sigma-Aldrich) in DI water (*27*, *28*). The soft NAL dECM-based nanofibrous scaffold electrospinning solution was prepared from 4% (w/v) soft MeHA, 2% (w/v) dECM, 2% (w/v) PEO, and 0.5% (w/v) Irgacure 2959 in DI water (*27*, *28*). Phase 1 and phase 2 scaffolds were fabricated by electrospinning using an 18-gauge metal needle (Hamilton) with a voltage of 17 kV onto a grounded collector with a voltage of −3 kV (*27*, *28*). The distance between the needle and collector was 15 cm, and the flow rate was 1 ml/hour. Scaffolds were purged with nitrogen and photocrosslinked with UV light (1.7 mW/cm^2^, 365 nm, UVP/Analytik Jena) for 20 min.

### Fabrication of 3D porous POMaC/BG scaffolds

POMaC was synthesized via condensation polymerization of 1,8-octanediol, citric acid, and maleic anhydride at 140°C under a nitrogen atmosphere for 3 hours, yielding pre-POMaC containing vinyl groups for further crosslinking (*32*). POMaC was covalently crosslinked with MeHA through free radical polymerization. Briefly, the polymers were combined with a photoinitiator (Irgacure 2959) and exposed to UV light to generate free radicals, triggering double bond cleavage and subsequent polymer crosslinking. This reaction resulted in the formation of a hybrid, crosslinked polymer network. Phase 3 scaffolds were composed of POMaC and BG (0/40 wt %, Mo-Sci Corporation). The resulting POMaC prepolymer was dissolved in 1,4-dioxane and mixed with BG (0/40 wt %, Mo-Sci Corporation) and sodium chloride (92 wt %; Thermo Fisher Scientific). The mixture was then molded and thermally crosslinked at 80°C for 7 days. After crosslinking, the scaffolds were immersed in DI water to remove the sodium chloride and subsequently dried by lyophilization for future use.

### Scaffold characterizations

dECM-based nanofibrous scaffolds and POMaC/BG scaffold were coated with iridium by a sputter coater (Quorum Q150T ES). A scanning electron microscope (FEI Quanta) was used to characterize the scaffold morphology and porosity. E-SEM (TFS Quanta 600) was used to observe the hydrated nanofibers. To evaluate the mineralization capacity of the scaffold surface, POMaC/0 BG and POMaC/40 BG scaffolds were immersed into a SBF, designed to mimic the human blood plasma (*60*), for up to 28 days. To assess dECM preservation within the treated bovine tissue, 100-μm-thick sections of native bovine ECM and bovine dECM were stained with H&E for nuclei visualization, Alcian Blue for GAG detection, and PSR for collagen distribution (*27*, *61*). To evaluate dECM distribution and preservation within the electrospun nanofibrous scaffolds, dry stiff AL, dry soft AL, and dry control scaffolds were stained under static conditions using PSR for collagen visualization, Alcian Blue for GAG detection, and DTAF for overall protein visualization (*27*, *61*). Staining was performed immediately following scaffold fabrication. Representative images were subsequently acquired using a Nikon wide-field microscope to assess collagen distribution, GAG preservation, and overall protein preservation within the electrospun scaffold matrix.

### Mechanical testing

Uniaxial tensile tests were performed on soft and stiff dECM/MeHA scaffold strips (width = 5 mm, length = 30 mm) using the Instron 5542 with a loading strain rate of 0.2%/s. Scaffold thickness was measured using a customized system described in a previous study (*27*). To standardize elastic modulus determination, stress-strain data were analyzed using a continuous exponential-linear curve-fitting algorithm that identifies the linear elastic region, and the elastic modulus was calculated from the slope of this region and applied uniformly across all samples (*27*, *62*). Compressive moduli of the nanofibrous scaffolds were measured using atomic force microscopy (AFM) (*63*). Before analysis, scaffolds were hydrated in PBS for at least 20 min (*64*). Scaffold surfaces were indented with a silicon nitride cantilever equipped with a three-sided pyramidal tip (catalog no. BL-AC40TS-C2, Asylum Research; spring constant: 0.09 N/m; tip radius: 8 nm) (*65*). Measurements were performed in contact mode using an NX12 AFM system (Park Systems) mounted on a Nikon ECLIPSE Ti2 inverted microscope (*66*). Force-distance curves were collected from multiple locations across the scaffold surface and analyzed using XEI software (Park Systems) to determine compressive moduli (*65*, *66*). Reported modulus values represent averaged measurements across the scaffold surface rather than individual electrospun fibers.

### FTIR and accelerated degradation testing

FTIR was acquired on a Thermo Electron Corp Nicolet 380 FTIR Spectrometer with 64 scans. The spectra were normalized at 1723 cm^−1^ for a better comparison. The accelerated degradation rate of porous scaffolds was assessed in vitro in PBS at 37°C for up to 56 days under static conditions. PBS was changed every week to maintain the pH above 7. At the predetermined time point, the samples were washed with DI water and lyophilized. The weight loss was calculated by comparing the initial weight (*W*_0_) with the weight measured at 0, 14, 28, and 56 days (*W*_t_). The degradation percentage was calculated using the following formula: Mass loss (%) = (*W*_0_ − *W*_t_)/*W*_0_ × 100%.

### Cell isolation and culture

Primary bTCs and bMSCs were isolated from the Achilles tendons and tibiae of juvenile bovine donors (approximately 3-month-old juvenile bovine donors, Research 87). The basal cell culture medium was prepared using high-glucose Dulbecco’s minimum essential medium (Gibco) with 10% fetal bovine serum (R&D Systems) and 1% penicillin-streptomycin solution (Corning). Cells were collected and seeded on the scaffolds after reaching 90% confluency rate.

### Immunofluorescence staining

After 3 days of culture on dECM-based nanofibrous scaffolds, passage 1 bMSCs were fixed with 4% paraformaldehyde at room temperature and blocked with 1% bovine serum albumin (Sigma-Aldrich). To assess YAP nuclear localization, samples were incubated with anti-YAP mouse monoclonal immunoglobulin G2a (IgG2a) antibody (1:200, Santa Cruz Biotechnology) followed by Alexa Fluor 546 goat anti-mouse IgG antibody (1:200, Life Technologies Corporation). The cytoskeleton was stained with Alexa Fluor 488 phalloidin (Life Technologies Corporation) at 37°C for 1 hour, and cell nuclei were stained by 4′,6-diamidino-2-phenylindole (Invitrogen). For transcriptional activation analysis, acetyl-H3K9 (H3K9ac, Invitrogen) primary antibody was applied, followed by Alexa Fluor 488 goat anti-rabbit secondary antibody (Invitrogen) (*67*). Imaging was performed using a Leica fluorescence microscope, and cell morphology was quantified with Fiji software.

### Cell proliferation and gene expression on scaffolds

Passage 1 bTCs or bMSCs were seeded at 5000 cells per scaffold (*n* = 5 per group). Cell proliferation was assessed by adding 440 μl of Cell Counting Kit-8 reagent (Dojindo, 1:10 dilution) to each scaffold and incubating at 37°C for 4 hours under 5% CO_2_. Absorbance was measured at 450 nm using a BioTek Synergy H1 microreader.

mRNA was isolated from cells using the TRIzol (Thermo Fisher Scientific)/chloroform (Thermo Fisher Scientific) extraction method (*57*). Total RNA concentrations were measured using a NanoDrop spectrophotometer (NanoDrop Technologies) (*68*). cDNA was synthesized using the PhotoScript II First-Strand cDNA Synthesis Kit (New England Biolabs). RT-qPCR was performed using a QuantStudio 6 Pro (Applied Biosystems) and Fast SYBR Green Master Mix (Applied Biosystems). The expression levels of collagen type I alpha 2 chain (*COL1A2*), collagen type II (*COL2*), collagen type III alpha 1 chain (*COL3A1*), tenascin-C (*TNC*), tenomodulin (*TNMD*), SOX-9SRY-box transcription factor 9 (*SOX-9*), transforming growth factor–β (*TGF-β*), and aggrecan (*ACAN*) were measured. Expression levels were normalized with respect to the housekeeping gene glyceraldehyde 3-phosphate dehydrogenase. Primer sequences are listed in table S1.

### RNA sequencing

RNA-seq was conducted on passage 1 bMSCs cultured on dECM/MeHA nanofibers with different stiffness and alignment configurations: stiff AL, stiff NAL, soft AL, and soft NAL. Total RNA was extracted using the TRIzol (Thermo Fisher Scientific)/chloroform (Thermo Fisher Scientific) method. RNA-seq library preparation, including ribosomal RNA depletion and 150-bp paired-end sequencing, was performed at Genewiz (South Plainfield) (*57*).

Sequence reads were processed with Trimmomatic v.0.36 for adapter and low-quality nucleotide removal then aligned to the *B. taurus* reference genome (Ensembl) using the STAR aligner v.2.5.2b (*57*). Unique gene hit counts were generated via FeatureCounts from the Subread package v.1.5.2. Differential expression analysis was performed in R using DESeq2 (*57*). Differential gene expression was assessed using a likelihood ratio test, with statistical significance defined as *P* < 0.05 (*57*). Differentially expressed genes were identified on the basis of an adjusted *P* value < 0.05 (Benjamini-Hochberg) and |log_2_ (fold change)| > 1.0 (*57*).

### Rabbit rotator cuff in vivo study design

The BMS was fabricated using a sequential electrospinning and simultaneous crosslinking strategy to ensure robust interfacial integration and prevent delamination. The assembly was performed in three steps: First, the soft NAL dECM-based nanofibrous layer was directly electrospun onto the preformed POMaC/40 BG porous scaffold for 6 hours to establish the initial biphasic interface. Second, immediately following this step, the stiff AL dECM-based nanofibrous layer was electrospun onto the uncrosslinked soft NAL layer for an additional 6 hours, thereby generating the complete multiphasic architecture. Both nanofibrous layers were maintained in an uncrosslinked state during deposition to facilitate fiber interpenetration across phase boundaries. Last, the fully assembled construct (stiff AL-soft NAL-POMaC/40 BG) was subjected to UV irradiation (365 nm, 1.7 mW/cm^2^, UVP/Analytik Jena) for 20 min. This final crosslinking step chemically and physically locked the phases together, resulting in a stable, integrated BMS. To assess scaffold stability and interfacial integrity, assembled BMS constructs were incubated in basal media for 7 days. Gross morphology, PSR staining, and DTAF staining were evaluated at days 0, 1, and 7 to identify potential delamination, phase separation, or loss of dECM components.

All in vivo procedures were approved by the University of Pennsylvania Institutional Animal Care and Use Committee (#806603). Each rabbit’s left shoulder served as the control (native ctrl), while the right shoulder underwent either BMS implantation [(+) BMS] or negative control [(−) BMS] surgical treatment. Rabbits were divided into two age groups: young (<10 months old) and old (>3 years old). For the (+) BMS treatment, the right shoulders of seven young and four old rabbits were used, while the (−) BMS treatment was applied to the right shoulders of four young and four old rabbits. The left shoulders of all rabbits were collected as part of the positive control group.

This study was reviewed and approved by the Institutional Animal Care and Use Committee of the University of Pennsylvania. Skeletally mature, female, New Zealand White rabbits were enrolled in the study. The animals were sourced from a commercial supplier (Charles River Laboratories). Animals received a subcutaneous injection of preanesthetic and general anesthesia consisting of ketamine (35 mg/kg) and xylazine (5 mg/kg). Following endotracheal intubation, animals were maintained under isoflurane/oxygen for the duration of the procedure. Using an aseptic technique, the right supraspinatus muscle and tendon served as the experimental side in all animals, with the left shoulder as an unoperated control. Briefly, an open craniolateral approach was performed on the right shoulder, approaching the supraspinatus muscle via splitting of the deltoid muscle followed by sharp transection of the right supraspinatus tendon from the insertion at the greater tuberosity. Two bone tunnels were drilled on both the supraglenoid and greater tubercle using 1-mm Kirschner wires. Bone troughs perpendicular to the direction of the supraspinatus muscle fiber on both the medial margin of the greater tubercle and the dorsal aspect of the glenoid were created. The tendon of the supraspinatus was secured using a modified Mason-Allen suture enhancement technique, followed by passing two strands each of #1PDS (Ethicon) through each bone tunnel and tying at the level of the greater tubercle. One strand of suture of each bone tunnel was knotted in a parallel pattern. The remaining strand of suture of one bone tunnel was knotted with the strand of another bone tunnel in a crossed pattern.

To evaluate the in vivo performance of the BMS, BMS scaffolds were placed between the tendon and bone [BMS-treated group: (+) BMS]. Control groups included unoperated rabbit shoulders (native Ctrl) and injured shoulders repaired using standard suture-based fixation without implantation of the BMS scaffold [(−) BMS]. Thus, the (−) BMS group served as a repair-only control and was not intended to represent an anchor-only control condition (Fig. 6C). The (+) BMS group included seven young and four adult right shoulders; the (−) BMS group included four young and four adult right shoulders. The left shoulders served as native controls. The deltoid and skin incision were closed in layers with 2-0 Monocryl (Ethicon). Postoperatively, animals were allowed free cage activity. During the postoperative time, clinical parameters including appetite, activity, signs of infection, bleeding, and wound dehiscence were evaluated daily by a veterinary surgeon. At 4 weeks postoperative, animals were euthanized with a commercially available euthanasia solution (pentobarbital 1 ml/5 kg) according to the guidelines set forth by the current American Veterinary Medical Association (AVMA) Panel on Euthanasia, and shoulders were harvested and processed for ex vivo analyses.

### Micro-CT scanning

Micro-CT scan was performed in Penn Center for Musculoskeletal Disorders MicroCT Imaging Core. Rabbit shoulders were fixed in 10% neutral buffered formalin for 4 days and then subjected to ex vivo micro-CT scanning using a Scanco μCT45 system (Scanco Medical AG). Scans were performed at an 18.5-μm voxel resolution with a 300-ms integration time, 145-μA current, and 55-kVp energy. A 2-mm-thick region of interest was selected from micro-CT images of each humerus bone to evaluate BV/TV, trabecular thickness, and Tb.sp.

### Histological analysis

After micro-CT scanning, rabbit shoulders were decalcified at room temperature using Formical-2000 for 3 weeks. After decalcification, the samples were dehydrated using graded ethanol (Decon Labs), embedded in paraffin (Azer Scientific), and sectioned into slices with a thickness of 8 μm by a rotary microtome (Leica). After sectioning, histological staining was conducted using H&E, Masson’s trichrome, Safranin O/Fast Green, and PSR staining (*27*, *57*). Polarizing light microscopy was carried out using a Leica DMLP Polarizing Light Microscope. The stained slices were imaged using a Zeiss Axiocam 506 mono camera, and images were processed with Zeiss ZEN 3.9 software.

### Statistical analysis

Experimental data were statistically analyzed using GraphPad Prism 9 (GraphPad Software). Before hypothesis testing, data normality and variance homogeneity were assessed using the Shapiro-Wilk test and the Brown-Forsythe test, respectively. For comparisons between two experimental groups, an unpaired Student’s *t* test was used for normally distributed data, whereas the Mann-Whitney *U* test was applied for nonparametric data. For multigroup comparisons, one-way analysis of variance (ANOVA) followed by Tukey’s post hoc test was performed. For datasets exhibiting heteroscedasticity or unbalanced sample sizes, Welch’s ANOVA with Games-Howell or Dunnett’s T3 multiple comparisons test was applied. Data are presented as means ± SD or SEM, as indicated in the figure legends. Statistical significance was defined as *P* < 0.05.
