# Supplementary material for: Engineering a biomimetic multiphasic suture anchor system for enhanced rotator cuff enthesis regeneration
Source: Sci Adv. 2026 Aug 21;12(34):eaea3128. doi: 10.1126/sciadv.aea3128 (PMC13496182; doi:10.1126/sciadv.aea3128)
Supplement: Supplementary file 1 [file sciadv.aea3128_sm.pdf]

Science Advances
AAAS

Supplementary Materials for

Engineering a biomimetic multiphasic suture anchor system for enhanced rotator cuff enthesis regeneration

Zizhao Li *et al.*

Corresponding author: Su Chin Heo, heosc@pennmedicine.upenn.edu

Sci. Adv. **12**, eaea3128 (2026)
DOI: 10.1126/sciadv.aea3128

This PDF file includes:

Figs. S1 to S5
Tables S1 and S2

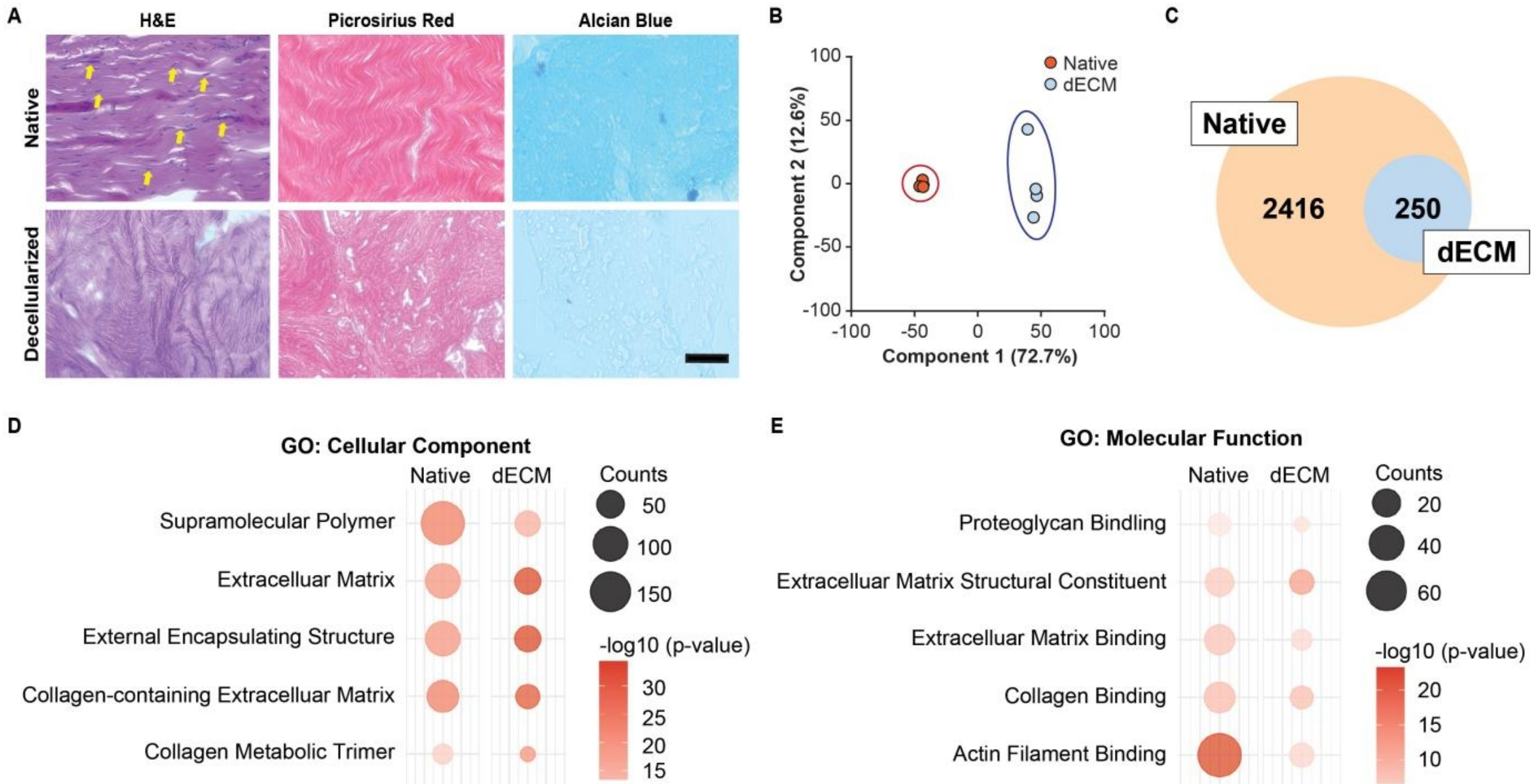

Figure S1. (**A**) Representative H&E, Picrosirius Red, and Alcian Blue staining of native and decellularized ECM tissues (Arrows indicate nuclei, scale bar = 50 μm). (**B**) Principal component analysis (PCA) showing distinct clustering of native and dECM samples. (**C**) Venn diagram illustrating the overlap of detected proteins between native and dECM groups. (**D**) Gene Ontology (GO) analysis of Cellular Component categories. (**E**) GO analysis of Molecular Function categories.

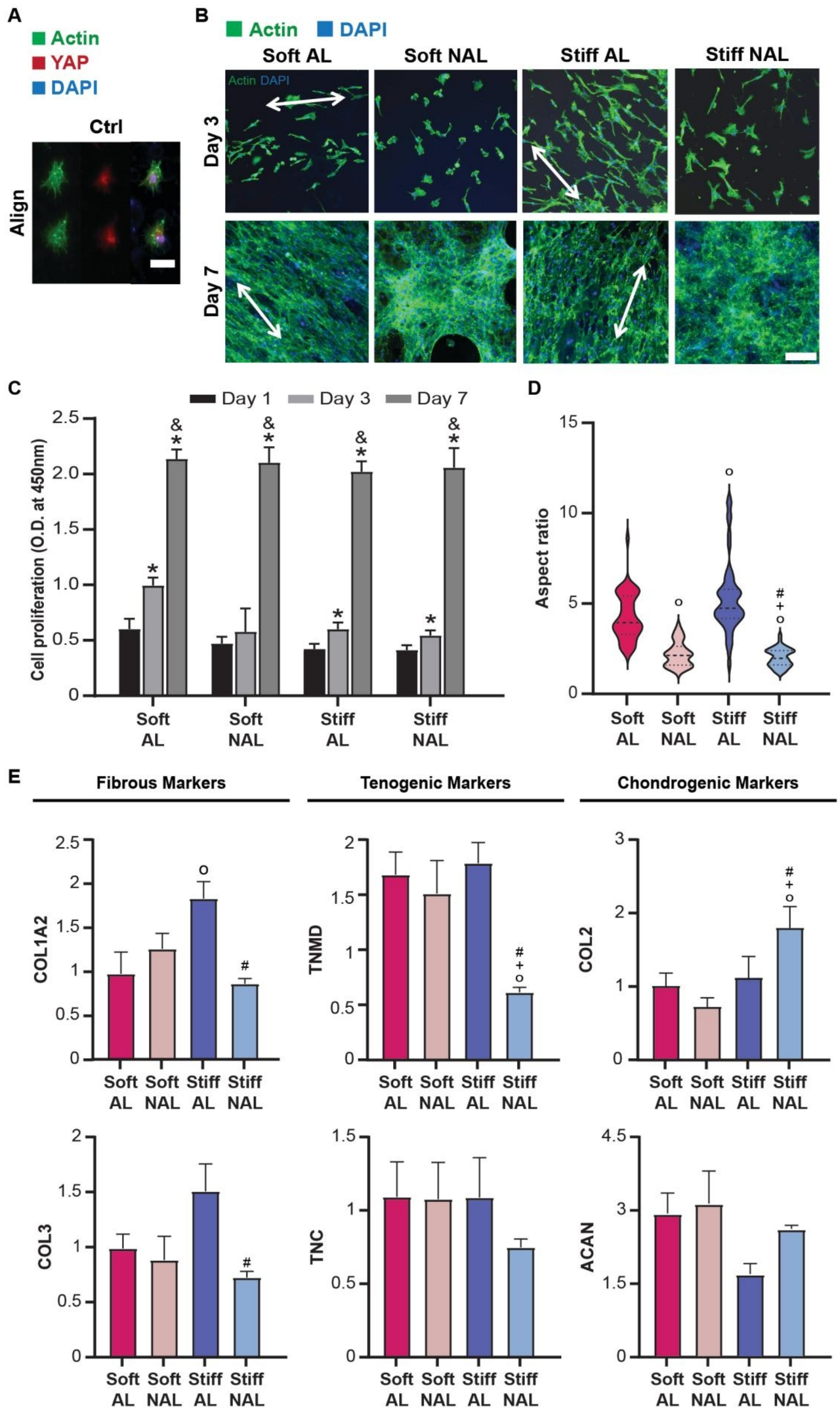

Figure S2. (**A**) Representative images of bMSCs cultured on aligned 100 % modified MeHA-

only scaffolds (Ctrl) for 3 days (Green: F-actin, Red: YAP, Blue: DAPI; Scale bar: 100 μm). (**B**) Representative bTC images cultured for 3 and 7 days (Allows: fiber direction; Green: F-actin, Blue: DAPI; Scale bar: 600 μm). (**C**) bTC proliferation determined by CCK8 assay (*: $p < 0.05$ vs. Day 1, &: $p < 0.05$ vs. Day 3, n = 5/group, means ± SD). (**D**) Quantification of bTC aspect ratios (○: $p < 0.001$ vs. Soft AL, +: $p < 0.001$ vs. Soft NAL, #: $p < 0.001$ vs. Stiff AL, n = 50, means ± SEM). (**E**) bTC gene expression at Day 7 (○: $p < 0.05$ vs. Soft AL, +: $p < 0.05$ vs. Soft NAL, #: $p < 0.05$ vs. Stiff AL, n = 5, means ± SEM).

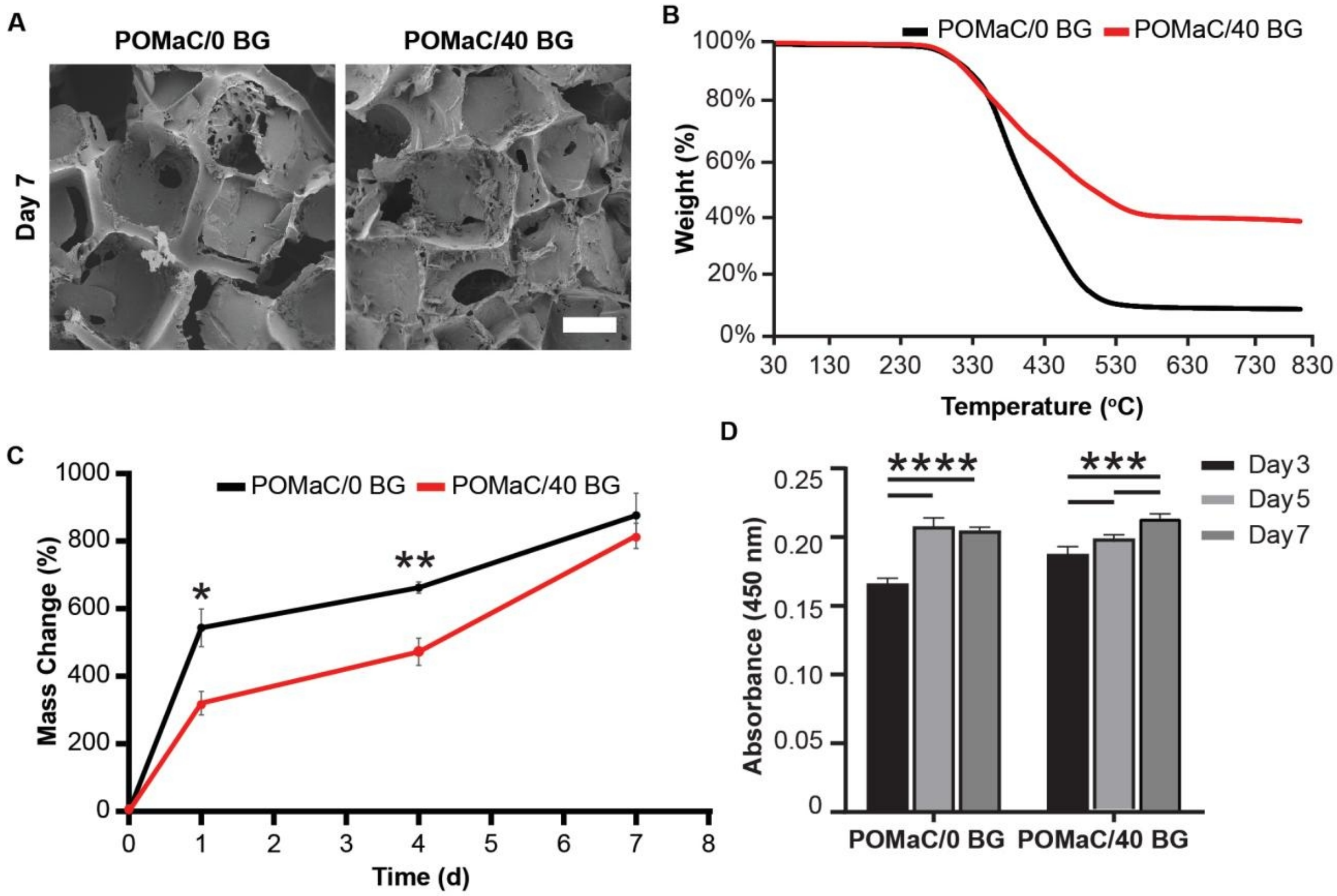

Figure S3. (**A**) SEM images of POMaC/0BG and POMaC/40BG scaffolds on Day 7, showing surface morphology and mineral deposition (scale bar = 200 µm). (**B**) Thermogravimetric analysis (TGA) of POMaC/0BG and POMaC/40BG scaffolds conducted under nitrogen atmosphere from 30°C to 800°C, showing thermal stability and inorganic content. (**C**) Swelling behavior of scaffolds with significantly higher swelling observed in POMaC/40BG (*: $p < 0.05$ at Day 1, **: $p < 0.01$ at Day 4, n = 4/group, means ± SEM). (**D**) bMSC proliferation assessed via CCK-8 assay, showing enhanced cell growth on POMaC/40BG scaffolds (***$p < 0.001$, ****$p < 0.0001$, n = 5/group, means ± SEM).

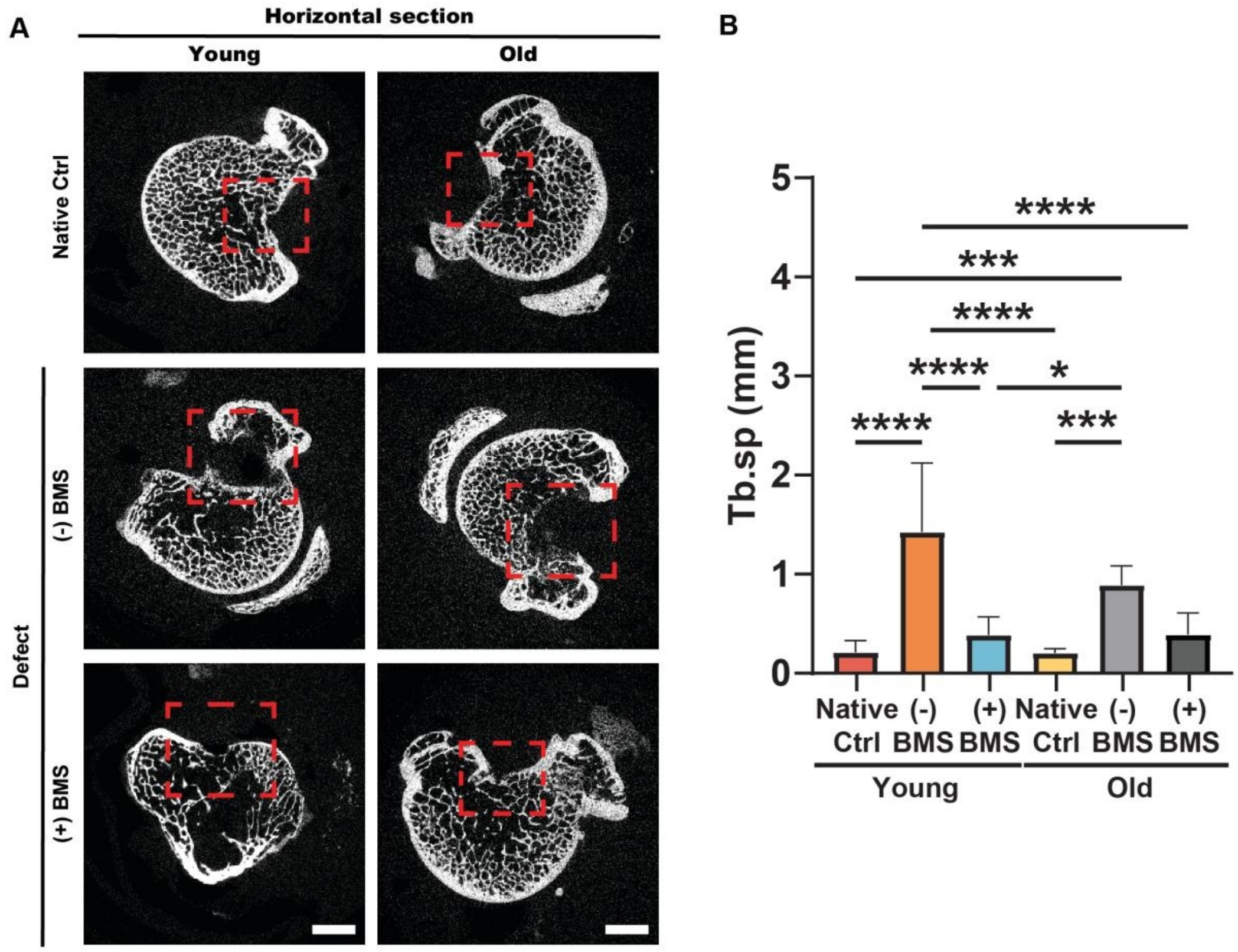

Figure S4. (**A**) Horizontally sectioned micro-CT imaging at 4 weeks post-operation, comparing Native Ctrl (native control), [(-) BMS] (defect), and [(+) BMS] (treated) groups, with highlighted regions of interest. (**B**) Quantitative analysis of the trabecular separation (Tb.Sp) in the regions of interest. Significant differences were observed among groups (n = 4–7; *: $p < 0.05$, ***: $p < 0.001$, ****: $p < 0.0001$, means ± SD).

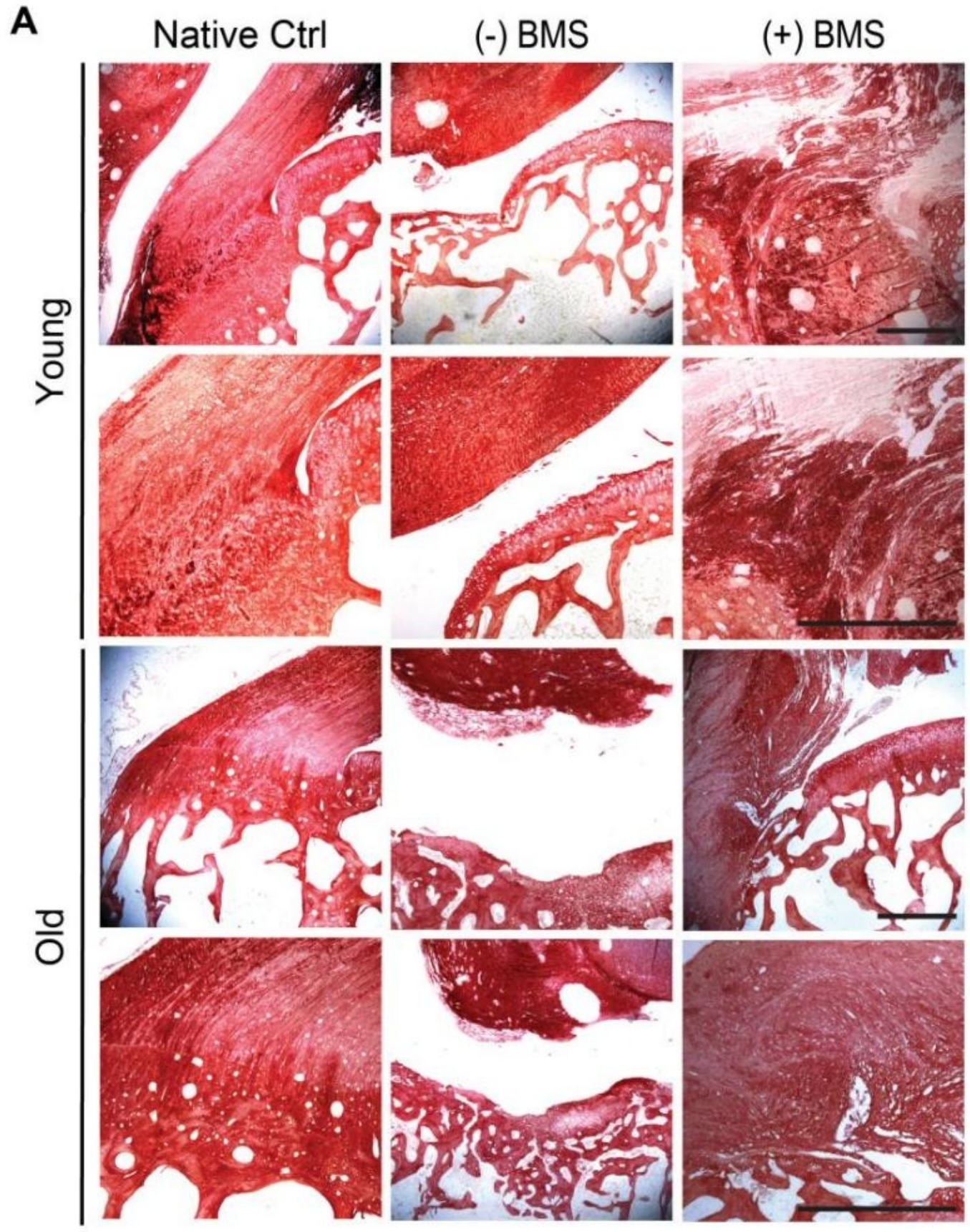

Figure S5. Picrosirius Red–stained sections of the tendon-to-bone interface at 4 weeks post-operation. Images were acquired using the widefield function of a Leica DMLP polarizing light microscope.

Table S1. Bovine primers used for RT-qPCR.

**Name**	**Gene**	**Primer Sequence**
Collagen type I alpha 2	*COL1A2*	F: 5’-AATTCCAAGGCCAAGAAGCATG-3’<br>R: 5’-GGTAGCCATTTCCTTGGTGGTT-3’
Collagen type II	*COL2*	F: 5’-AAGAAGGCTCTGCTCATCCAGG-3’<br>R: 5’-TAGTCTTGCCCCACTTACCGGT -3’
Collagen type III	*COL3*	F: 5’-CTGACATTTAGACATGATGAG-3’<br>R: 5’-ACTGACCGAGATGGGAGCAT-3’
Tenascin-C	*TNC*	F: 5’-CCTGGAATCTCCACGGAGTG-3’<br>R: 5’-GAACCTCACAGTGACCAGGG-3’
Tenomodulin	*TNMD*	F: 5’-TCTGGCGTGACGGGTCTT-3’<br>R: 5’-AAAAAAGGCATTGAACAAAACGA-3’
Aggrecan	*ACAN*	F: 5’-CCTGAACGACAAGACCATCGA-3’<br>R: 5’-TGGCAAAGAAGTTGTCAGGCT-3’
SRY-box transcription factor 9	*SOX-9*	F: 5’-TGAAGAAGGAGAGCGAGGAG-3’<br>R: 5’-GTCCAGTCGTAGCCCTTGAG-3’
Transforming Growth Factor Beta	*TGF-β*	F: 5’-CACGTGGAGCTGTACCAGAA-3’<br>R: 5’-ACGTCAAAGGACAGCCACTC-3’
Glyceraldehyde 3-phosphate dehydrogenase	*GAPDH*	F: 5’-ATCAAGAAGGTGGTGAAGCAGG-3’<br>R: 5’-TGAGTGTCGCTGTTGAAGTCG-3’

Table S2. Modified histological scoring system for assessing rotator cuff enthesis regeneration.

Category	Score 1	Score 2	Score 3	Score 4
**Collagen Density at Enthesis**	Minimal	Sparse	Moderate	Dense
**Fiber Maturity and Thickness**	Poor	Partial	Developed	Well-developed
**Collagen Fiber Orientation**	Random	Partial aligned	Moderately aligned	Well-aligned
**Tendon-Bone Structural Integrity**	No transition	Abrupt	Partial gradient	Gradual gradient
**Enthesis Insertion Continuity (%)**	<25	26-50	51-75	>75
**Cellularity at the Tendon–Bone Interface**	Acellularity	Low cellularity	Moderate cellularity	High cellularity
